# Comparison of Aroma and Taste Profiles of Pixian Douban Fermented with Traditional Open Process or Industrial Closed Process

**DOI:** 10.3390/foods15132384

**Published:** 2026-07-03

**Authors:** Qiuyu Lan, Chenglin Zhu, Peiyi Wang, Luca Laghi

**Affiliations:** 1 Modern Industrial College of Traditional Chinese Medicine and Health, Li Shui University, Lishui 323000, China; lanqiuyu211@gmail.com; 2 Department of Agricultural and Food Sciences, University of Bologna, 47521 Cesena, Italy; 3 College of Pharmacy and Food, Southwest Minzu University, Chengdu 610041, China; chenglin.zhu@swun.edu.cn (C.Z.); 202330903050@stu.swun.edu.cn (P.W.)

**Keywords:** flavor, broad bean paste, closed fermented Doubanjiang, open fermented Doubanjiang, sensory evaluation

## Abstract

Pixian Doubanjiang (PXDB) is a traditional Chinese fermented condiment whose characteristic aroma and taste are strongly influenced by fermentation conditions. This study aimed to systematically compare the flavor profiles of PXDB produced via traditional open fermentation (TOF) and industrial closed fermentation (ICF), aiming to elucidate the chemical basis of sensory divergence and provide scientific support for industrial process optimization. In this study, PXDB samples were evaluated using sensory evaluation, GC-IMS, ^1^H-NMR metabolomics, and multivariate statistical analysis. Sensory evaluation revealed that ICF exhibited a stronger soy sauce-like aroma, alcohol note, and umami intensity, whereas TOF and ICF showed comparable sweetness, sourness, chili-like aroma, and roasted aroma. GC-IMS putatively identified 126 volatile compounds, and multivariate analyses demonstrated a clear separation between the two fermentation modes. Based on combined criteria of VIP > 1, FDR-adjusted *p* < 0.05, and |fold change| > 2, 35 differential volatile compounds were identified. ICF was characterized by higher levels of esters, particularly ethyl esters, and selected ketones, while TOF showed enrichment of higher alcohols, terpenes, and sulfur compounds. ^1^H-NMR analysis identified 54 non-volatile metabolites, of which 16 differed significantly between TOF and ICF, mainly involving amino acids, organic acids, and carbohydrates. Pathway analysis highlighted branched-chain amino acid and glutamate-related metabolism as key contributors to flavor divergence. Correlation analysis further revealed that soy sauce-like aroma and umami perception were strongly associated with amino acid-derived metabolites, esters, sulfur-containing compounds, and branched-chain aldehydes, highlighting the coordinated contribution of volatile and non-volatile compounds to flavor differentiation. Overall, fermentation mode was found to reshape PXDB flavor through coordinated modulation of volatile and non-volatile metabolism, providing experimental insight for improving industrial fermentation quality.

## 1. Introduction

Pixian Douban (PXDB), a representative fermented chili–broad bean paste originating from Sichuan Province, is widely recognized as “the soul of the Sichuan cuisine” [1]. Pixian Doubanjiang is a protected geographical indication (GI) product in China and represents a key element of traditional Sichuan culinary culture. The PXDB industry has developed rapidly in recent years, with an annual production exceeding 1,100,000 tons and a market value of approximately 1170 billion CNY. PXDB fermentation is a complex and multi-stage process involving chili fermentation, meju (dried fermented broad beans) fermentation, and subsequent mixed aging, during which diverse microbial communities transform raw materials into a wide range of flavor compounds [2]. In practice, PXDB fermentation includes diverse strategies such as natural fermentation, semi-controlled fermentation with inoculation, and laboratory-scale temperature-controlled strategies [2,3]. Traditional production mainly relies on natural fermentation, where microorganisms originate from raw materials and the environment, leading to rich but variable product quality [4]. As consumer demand for stable quality and industrial-scale production continues to rise, PXDB manufacturing has undergone a transition from traditional open fermentation (TOF) to industrial closed fermentation (ICF). However, whether these two fermentation systems produce comparable aroma and taste characteristics remains unclear, and systematic flavor-based comparisons are still limited.

The quality of PXDB is influenced by multiple environmental factors, including temperature, oxygen availability, sunlight exposure, and moisture content [4,5], all of which differ substantially between the two fermentation approaches. The traditional fermentation technology of PXDB is typically performed in outdoor or semi-outdoor strip pools or ceramic vats, allowing the mash to be fully exposed to sunlight, oxygen, natural microbial communities, and fluctuating environmental conditions. This open system often leads to strong but batch-dependent flavor characteristics and risks linked to food safety, making consistent industrial production challenging [4]. In contrast, the closed fermentation in tank fermenters effectively avoids weather-dependent fluctuations and reduces contamination risks, resulting in more standardized fermentation kinetics and stable product quality [6]. Ding et al. [4] compared the volatile profiles of doubanjiang-meju produced by industrial closed fermentation and traditional open fermentation, reporting that the closed fermentation products exhibited more desirable volatile characteristics than those obtained from the open fermentation process. Despite the importance of fermentation mode on product quality, comparative studies examining the flavor profiles of PXDB produced via traditional open fermentation and industrial closed fermentation remain scarce [4,5,6].

Flavor, encompassing taste, aroma, and chemical composition, is a pivotal characteristic that aptly mirrors the quality of PXDB, a product renowned for its deep red color, characteristic umami-rich taste, and harmonious profile of spicy, salty, and fermented aromas [3,7,8]. The aroma profile of PXDB consists of abundant aroma-active molecules such as esters, aldehydes, alcohols, acids, pyrazines, and sulfur compounds [4,9]. During fermentation, macromolecules such as proteins, carbohydrates, and lipids undergo enzymatic degradation and metabolic conversion, generating a diverse array of volatile and non-volatile compounds. These biochemical transformations are fundamental to the development of PXDB flavor, linking microbial activity with the formation of aroma and taste attributes. Meanwhile, non-volatile taste-related metabolites, including amino acids, peptides, organic acids, and sugars, are gradually accumulated as fermentation progresses [8]. Despite previous studies investigating volatile compounds or microbial characteristics of PXDB [2,3,4,5,7], most have focused on single fermentation stages or controlled fermentation on a laboratory scale. Given the complexity of PXDB flavor, investigating the flavor profile of PXDB fermented with ICF and TOF requires a comprehensive approach that considers both volatile and non-volatile compounds.

Given the differences in environmental conditions and microbial exposure between TOF and ICF, it is hypothesized that ICF could yield PXDB with more consistent and reproducible flavor profiles, whereas TOF may generate more variable but potentially richer aroma and taste characteristics. The present study aims to systematically elucidate the volatile and non-volatile metabolic profiles of these fermentation modalities, identify the key metabolic pathways and chemical shifts that underpin the observed sensory divergence, and provide actionable scientific recommendations for optimizing industrial fermentation strategies. To enable a controlled comparison of fermentation setups, the two production methods were implemented within a single production system using the same batch of raw materials and identical formulation. GC–IMS and ^1^H-NMR-based metabolomics were applied to characterize volatile and non-volatile profiles, respectively, combined with multivariate statistical analysis. The findings of this work are expected to provide scientific support for improved process control, quality consistency, and industrial upgrading while preserving the traditional flavor characteristics of this iconic fermented food.

## 2. Materials and Methods

### 2.1. Ethical Considerations

This study involved sensory evaluation by trained panelists employed by the collaborating company. According to institutional guidelines, formal ethical review and approval were waived because the study involved the evaluation of food products and did not require the collection of any personal, medical, or sensitive data. Participation was entirely voluntary, and all panelists provided informed consent prior to the sensory evaluation.

### 2.2. PXDB Samples

Two kinds of PXDB samples were provided by FanSaoGuang Food Co., Ltd. (Chengdu, China), a producer officially authorized to use the PGI certification for Pixian Doubanjiang. Samples were collected from an open strip fermentation pool and a closed tank fermenter, namely traditional open fermentation PXDB (TOF) and industrial closed fermentation PXDB (ICF). The fermentation process of PXDB in this study consisted of three stages, including meju fermentation, chili fermentation, and subsequent mixed fermentation. For the meju fermentation, broad beans were mixed with wheat flour and starter culture at a ratio of 1000:100:0.3 (*w*/*w*/*w*), and salt was added to achieve a final concentration of 15% (*w*/*w*). The starter culture is composed of a standardized microbial consortium, primarily including *Aspergillus oryzae* CICC 2339, *Aspergillus oryzae* ACCC30467, and *Aspergillus awamori* ACCC30368, provided by Shandong Hezhong Kangyuan Biotechnology Co., Ltd. (Linyi, China). The meju was fermented for 180 days. For the chili fermentation, fresh chili peppers (Honglong No. 13) were mixed with 15% (*w*/*w*) salt and fermented for 120 days. Afterward, the fermented meju and chili were combined and subjected to mixed fermentation for an additional 15 days to obtain the final PXDB product. A schematic diagram detailing the implementation of these two fermentation stages, including environmental parameters and vessel management, is provided in Figure S1.

All fermentation stages were conducted under the respective TOF or ICF conditions, differing primarily in fermentation setup, while other parameters were kept consistent. For each fermentation mode, samples were collected from independent fermentation vessels within the same production system. The samples of ICF were collected from the top and bottom of the fermenter after timely mixing while the samples of TOF were taken from the four vertices and the center of the open fermentation pool after timely mixing. The obtained samples sealed in sterile bags were transported to the laboratory immediately and stored in a refrigerator at −20 °C. The analysis of these samples was completed within a week.

### 2.3. GC-IMS Analysis

Volatile compounds were analyzed using a FlavourSpec^®^ gas chromatography–ion mobility spectrometry system (G.A.S., Dortmund, Germany) equipped with a CTC-PAL 3 static headspace autosampler (CTC Analytics AG, Zwingen, Switzerland). To ensure the reproducibility and statistical validity of the results, three independent biological replicates were analyzed for each experimental group, with samples collected from separate fermentation vessels. Briefly, 1.0 g of the sample was accurately weighed into a 20 mL headspace vial and incubated at 80 °C for 20 min with agitation at 500 rpm. Subsequently, 500 μL of headspace gas was injected in splitless mode, and each sample was analyzed in triplicate. The injector and syringe temperatures were maintained at 80 °C and 85 °C, respectively. Gas chromatographic separation was performed on an MXT-WAX column at a constant column temperature of 60 °C using high-purity nitrogen (≥99.999%) as the carrier gas. The carrier gas flow rate was initially set at 2.0 mL/min for 2 min, linearly increased to 10.0 mL/min over 8 min, then further increased to 100.0 mL/min within 10 min and held for 40 min, resulting in a total run time of 60 min. The drift tube was operated in positive ion mode with a tritium (^3^H) ionization source. The drift tube length was 53 mm, with an electric field strength of 500 V/cm and a tube temperature of 45 °C. Nitrogen (≥99.999%) was used as the drift gas at a constant flow rate of 75.0 mL/min.

For compound identification, a mixed standard solution containing six analytical-grade n-ketones, including 2-butanone, 2-pentanone, 2-hexanone, 2-heptanone, 2-octanone, and 2-nonanone, was analyzed to establish retention time and retention index (RI) calibration curves. Retention indices of target compounds were calculated and tentatively identified by comparison with the built-in GC RI database (NIST/EPA/NIH Mass Spectral Library (NIST 20), Gaithersburg, MD, USA, 2020) and IMS drift time library in VOCal software (version 0.4.10, G.A.S., Dortmund, Germany). All compound identifications in this GC-IMS dataset are classified as Level 3 tentative identifications following the standardized framework proposed by the Chemical Analysis Working Group Metabolomics Standards Initiative. The Reporter, Gallery Plot, and Dynamic PCA plugins in VOCal were employed to generate three-dimensional and two-dimensional spectra, difference plots, fingerprint maps, and principal component analysis (PCA) plots for comparative analysis of volatile profiles among samples.

### 2.4. ^1^H-NMR Analysis

The ^1^H-NMR analysis followed a previously established protocol [9]. Four independent biological replicates were analyzed for each experimental group, with the same modality as gas chromatography. Briefly, one gram of each fresh PXDB sample (water content around 55%) was mixed with 12 mL of a 7% (*w*/*w*) aqueous solution of trichloroacetic acid (TCA) for 20 s at 14,000 rpm with an Ultraturrax mincer (IKA, Staufen, Germany). The mix was then centrifuged at 18,630× *g* for 10 min at 4 °C. Then, 0.6 mL of the supernatant was added with 0.1 mL of a D_2_O solution of 3-(trimethylsilyl)-propionic-2,2,3,3-d_4_ acid sodium salt (TSP) 10 mmol/L for a final concentration of TSP equal to 1.43 mmol/L. The pH was adjusted to 7.00 ± 0.02 using 9 mol/L KOH in an Eppendorf microfuge tube. After centrifuging once more under the above conditions, 0.65 mL of the supernatant was transferred to an NMR tube for analysis. ^1^H-NMR spectra of the samples were acquired using a 600.13 MHz AVANCE III spectrometer (Bruker, Beijing, China) with a 5 mm BBO at 298 K. The CPMG pulse sequence was employed with suppression of the solvent signal, and the following parameters were set: fid size: 32 k, number of scans: 256, number of dummy scans: 16, spectral width: 12 ppm, acquisition time: 2.28 s, delay d1: 5 s, echo time: 400 µs, and number of echoes: 400. The NMR spectra were pre-processed and adjusted using Topspin 3.1, with TSP serving as a reference for spectrum calibration (δ = −0.017).

The concentration of each molecule was calculated from the area of one of its signals, shown in Figure S2 and described in detail in Table S4. Its area has been determined by global spectra deconvolution (GSD), using MestReNova software (version 14.2.0-26256, Mestrelab Research S.L., Santiago De Compostela, Spain). The performance of this algorithm is pictorially represented in Figure S3 in two key cases: one of severely superimposed signals and one of particularly crowded spectra portions. A line broadening of 0.3 and baseline adjustment using the Whittaker Smoother procedure were applied prior to GSD. To obtain the concentration of metabolites, while at the same time minimizing the effects of varying sample dilutions, a two-step approach, suggested by Zhu et al., was applied [10]. First, the ratio of the TSP peak area to the total spectral area was used to identify the sample with median water dilution. Metabolites’ concentrations in this reference sample were then determined using TSP as an internal standard. Probabilistic Quotient Normalization (PQN) [11] was subsequently applied to adjust the other spectra towards the reference, therefore obtaining concentrations adjusted for water dilution.

### 2.5. Sensory Evaluation

The sensory characteristics of two kinds of samples were evaluated by the quantitative description analysis (QDA). The senior staff coming from Sichuan Fansaoguang Food Co., Ltd. (Chengdu, China) were recruited as candidates. Firstly, the candidates were screened for their health status, sensory acuity and familiarity with PXDB by an initial recruitment questionnaire. Next, they were required to complete sensory ability tests for flavor identification in the allotted time and ranked according to the test results. At last, the candidates who achieved at least 70% acuity during the sensory ability tests were selected as the assessors. The group was composed of ten senior staff (five females and five males, aged 23–34). The sensory panel was selected, trained, and monitored in accordance with ISO 8586:2012 guidelines [12]. Firstly, the flavor description table was obtained according to the descriptors proposed by the evaluators. Secondly, the assessors were trained with flavor standards to reach agreement on the selected sensory descriptions and their intensities until all assessors could identify the provided standards correctly.

To eliminate bias and ensure independence, a double-blind procedure was employed. Specifically, the panelists were blind to the research objectives and the corresponding fermentation modes (TOF and ICF) of the samples. Sensory evaluation was performed in a sensory evaluation laboratory at 23 ± 2 °C, and 10 g of sample was taken into a 30 mL plastic cup and coded randomly. Before evaluating each sample, the evaluators washed their mouths with purified water. Sensory attributes, including soy sauce aroma, chili pepper-like aroma, alcohol-like aroma, roasted aroma, umami, sourness, sweetness, and aftertaste, were evaluated using a structured scoring system. Aftertaste was defined as the residual sensory perception remaining after swallowing, including both aroma persistence and taste duration, as commonly described in sensory evaluation studies. The intensity of aromas was expressed on a 0–9 scale (0 represented none, 9 represented very strong). The sensory evaluation of each sample was tested four times, and the averaged results were obtained [13].

### 2.6. Statistical Analysis

Statistical analysis was performed using the R (Ver. 4.0.2) computational language. To achieve normality, data distribution was transformed using Box and Cox’s method (Box & Cox, 1964 [14]). Differences in metabolite levels between PXDB produced by TOF and ICF were assessed using Student’s *t*-test, with *p*-values adjusted for multiple comparisons by the false discovery rate (FDR) method. To elucidate the overall trends in flavor and metabolomic profiles induced by different processing, principal component analysis (PCA) models and orthogonal projections to latent structures–discriminate analysis (OPLS-DA) models were performed using MetaboAnalyst 5.0 (https://www.metaboanalyst.ca). Prior to multivariate analysis, the metabolite was ignored to analyze when missing values accounted for more than 50% of the total data. Other missing values were filled with 1/5 of the minimum positive value and then normalized using PQN to correct for systematic variations, followed by auto scaling. The reliability of the OPLS-DA models and the risk of overfitting were rigorously evaluated using a 7-fold cross-validation procedure and a permutation test comprising 200 iterations. The quality of the models was assessed based on the predictive ability parameter (Q^2^ nad R^2^), ensuring that the observed group separations were statistically valid and not due to chance. The metabolic pathway analysis was conducted using the key differential metabolites identified by ^1^H-NMR using the MetaboAnalyst 6.0 platform (https://www.metaboanalyst.ca). Specifically, the metabolites meeting the criteria of VIP > 1.0 and FDR-adjusted *p* < 0.05 were selected for pathway analysis.

## 3. Results

### 3.1. Sensory Evaluation

**Figure 1:**
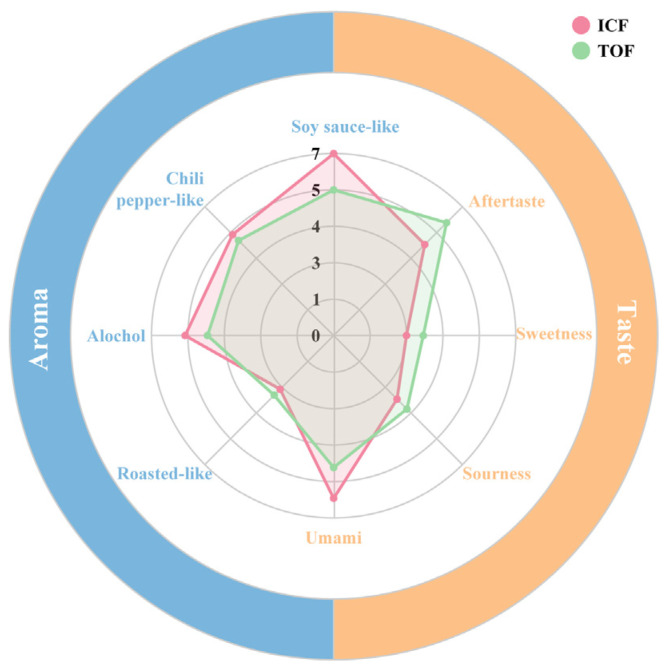
Radar plot of QDA sensory profiles of Pixian Doubanjiang produced by TOF and ICF. Aroma and taste attributes were evaluated by a trained sensory panel.

The special aroma and taste of PXDB were described by the QDA (Figure 1). The results were visualized in a radar plot based on the scores of the eight descriptors: soy sauce, chili pepper-like, alcohol, roasted smell, umami, sour, sweet, and aftertaste, as listed in Table S1. Compared with the TOF, the ICF showed stronger attributes of soy sauce, and umami (*p* < 0.05, FDR < 0.05). Conversely, the ICF and TOF had similar aroma profiles related to sweetness, sourness, chili pepper-like aroma and roasted aroma.

### 3.2. GC-IMS Analysis

To highlight differences in the flavor profiles of PXDB related to TOF and ICF, GC-IMS was employed. A total of 126 volatile features were detected, and subsets of these were tentatively assigned to specific compounds, including 16 alcohols, 20 aldehydes, 30 esters, 18 ketones, 16 terpenes, eight acids, eight sulfur compounds, and 10 miscellaneous compounds, as listed in Table S2. Additionally, single volatile compounds such as 2-methylpropanoic acid, butanoic acid, pentanoic acid, 1-hexanol, 1-pentanol, 3-methyl-1-butanol, 3-methyl-2-butenal, furfural, phenylaceraldehyde, propanal, ethyl 3-methylbutanoate, ethyl hexanoate, ethyl lactate, ethyl octanoate, 2-heptanone, acetoin, methional, and linalool oxide exhibited multiple spots or signals. These signals, labeled as -M and -D, were interpreted as monomers and dimers in the fingerprint region, respectively, displaying varying concentrations.

**Figure 2:**
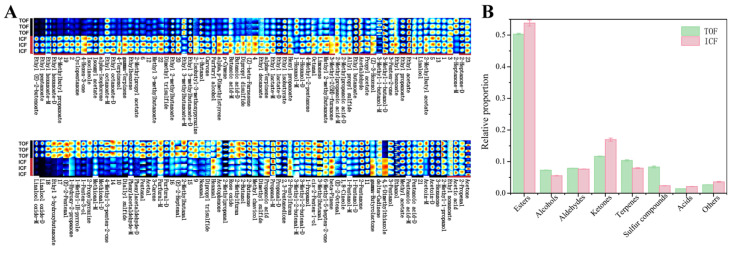
GC-IMS volatile fingerprinting and class distribution. (**A**) Fingerprint plot of volatile compounds in TOF and ICF samples. Each row represents a sample, and each column corresponds to a specific volatile compound. The color intensity represents the signal intensity of the compounds: blue indicates a low signal intensity, while the gradient from yellow to red signifies an increasing signal intensity. The labels of volatile compounds are listed in Table S2. (**B**) Relative proportion of different volatile compound classes (based on the number of compounds).

As presented in Figure 2A, the fingerprint analysis revealed a notable distinction between TOF and ICF, with varying signal intensities for each group. Blue indicates lower signal intensity, while the shift from yellow to red represents increasing concentrations of the detected volatile compounds. Several signal regions corresponding to alcohols, sulfur compounds, and ketones showed higher intensities in TOF, whereas ICF samples were characterized by stronger signals for alcohols and esters, especially ethyl esters. To better visualize these differences, the relative proportion of volatile compound categories were quantified and summarized as a bar chart (Figure 2B). The results showed that TOF samples exhibited higher levels of specific alcohols, terpenes, sulfur compounds, and ketones (FDR-adjusted *p* < 0.05), while ICF samples had significantly higher relative contents of multiple esters, ketones, and acids (FDR-adjusted *p* < 0.05). These differences contributed substantially to the overall discrimination between TOF and ICF, consistent with the clustering patterns observed in the GC-IMS fingerprint plot.

**Figure 3:**
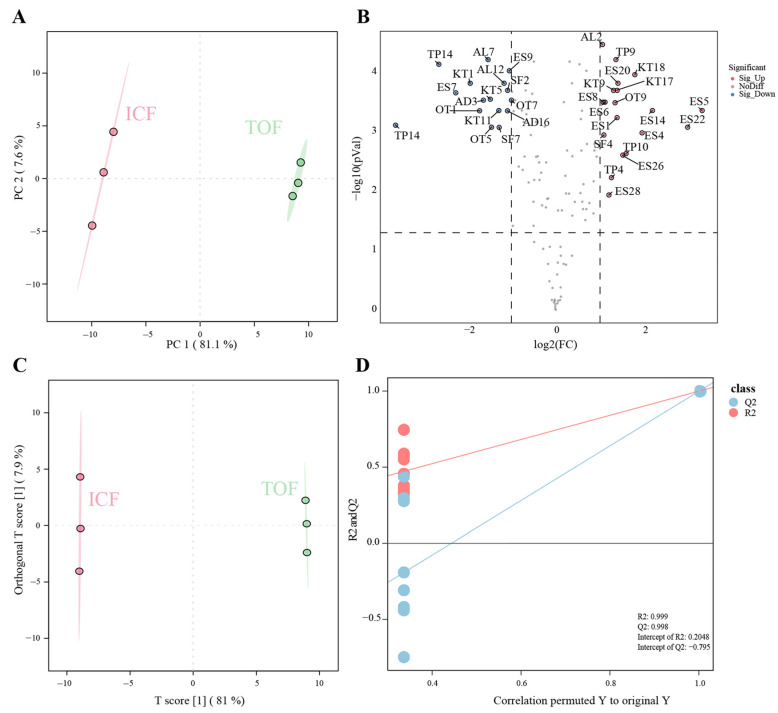
Multivariate statistical analysis of volatile compounds from PXDB samples fermented by ICF and TOF. (**A**) Score plot of PCA. (**B**) Volcano plot exhibiting the metabolites (*p* < 0.05 and |log_2_ (FC)| > 1) and −log_10_(*p* value); *p* value was adjusted with FDR. (**C**) Score plot of the OPLS-da model. (**D**) Permutation test of the OPLS-DA model (based on 200 simulations).

Multivariate statistical analyses were performed to evaluate the differences in volatile compounds between Pixian Doubanjiang samples fermented by ICF and TOF, as shown in Figure 3. A PCA score plot (Figure 3A) revealed a clear separation between ICF and TOF samples along the first two principal components, indicating distinct volatile profiles associated with different fermentation modes. Replicates within each group clustered closely, demonstrating good analytical reproducibility and sample consistency. OPLS-DA was subsequently applied to further examine the separation between the groups, as shown in Figure 3C. The OPLS-DA score plot demonstrated a distinct separation between ICF and TOF samples without any overlap, with satisfactory model parameters (R^2^X = 0.796, R^2^Y = 0.998, Q^2^ = 0.998). Model validation using a permutation test with 200 simulations showed that the original model had significantly higher R^2^ and Q^2^ values than those of the permuted models, and the Q^2^ regression line exhibited a negative intercept (Figure 3D), indicating good model robustness and the absence of overfitting. Based on the OPLS-DA model, a total of 87 volatile compounds displayed VIP values greater than 1, listed in Table S3. However, no compounds met the stricter VIP threshold of 1.2.

Considering the relatively dispersed contribution of individual variables, fold change was additionally introduced to further refine the selection of differentially abundant volatile compounds. A volcano plot was therefore constructed based on log_1.5_(ICF/TOF) and −log_10_(*p*-value), with *p*-values adjusted using the false discovery rate (FDR) method (Figure 3B). Using the combined criteria of VIP > 1, FDR-adjusted *p* < 0.05, and |fold change| > 2, a total of 35 volatile compounds were identified as significantly different between ICF and TOF. Among these compounds, 19 were significantly up-regulated and 16 were significantly down-regulated in ICF compared to TOF. These differentially abundant volatile compounds constituted the main contributors to the observed differences in volatile profiles between the two fermentation processes.

### 3.3. ^1^H-NMR Analysis

^1^H-NMR was applied to compare the water-soluble metabolome profiles of PXDB obtained by ICF and TOF. A total of 54 metabolites were identified and quantified (Table S4), encompassing amino acids and amines (18), alcohols (5), carbohydrates (7), nucleotides (4), organic acids (10), and miscellaneous compounds (10). To facilitate visual inspection of the signal assignments, Figures S4–S17 show selected portions of the spectra acquired in this study, superimposed with spectra of pure reference compounds. Since only the spectral regions containing the signals used for quantification are displayed, a complete representative spectrum in Chenomx format is also provided in the Supplemental Materials, together with a simulated spectrum showing the full spectral profiles of the quantified compounds.

Among these metabolites, 16 exhibited significant differences (FDR-adjusted *p* < 0.05) between TOF and ICF samples.

**Figure 4:**
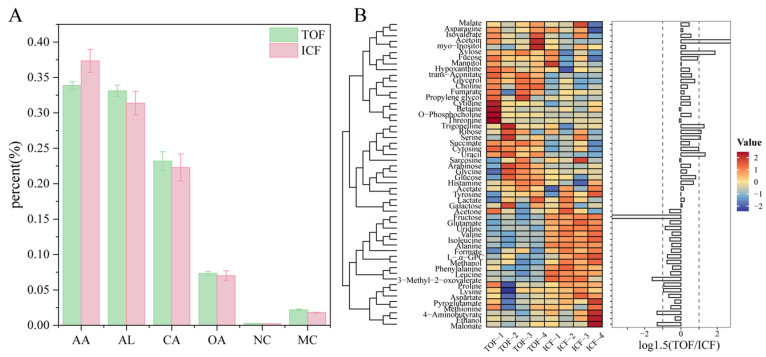
^1^H-NMR observation of PXDB by ICF and TOF. (**A**) Distribution of different kinds of metabolites in the samples; (**B**) Heatmap of contents of metabolites in two types of PXDB. A, amino acids, peptides and analogs; AL, alcohols; CA, carbohydrates; OA, organic acids; NC, nucleotides and nucleosides; MC, miscellaneous.

Figure 4A showed the proportions of metabolite categories in PXDB produced by ICF and TOF, with amino acids, alcohols, and carbohydrates exhibiting the highest relative abundance. Although ICF samples exhibited levels of amino acids relatively higher than TOF, no significant differences were observed among the six metabolite categories (FDR-adjusted *p* > 0.05). The heatmap visualization revealed distinct metabolite distribution patterns between the TOF and ICF samples (Figure 4B). To further quantify the differences, the right panel shows the log_1.5_(TOF/ICF) fold change of the individual metabolites. Positive values indicate enrichment in TOF, whereas negative values represent enrichment in ICF. TOF samples displayed higher abundances of numerous sugars (e.g., xylose, fucose, ribose), organic acids (e.g., malate, fumarate, succinate), and several amino acids. In contrast, the ICF samples were characterized by higher levels of metabolites such as fructose, 3-methyl-2-oxovalerate, 4-aminobutyrate, and malonate.

**Figure 5:**
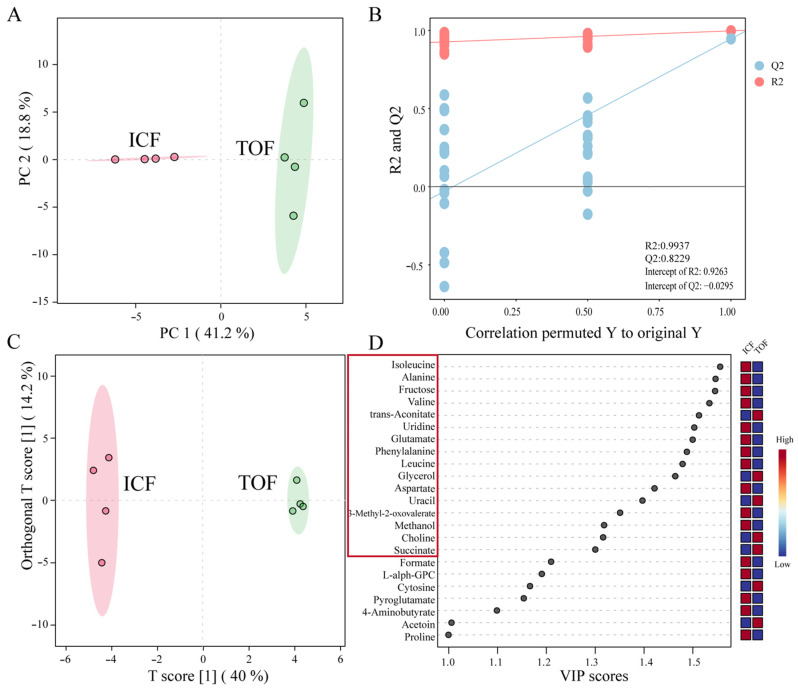
Multivariate statistical analysis of metabolomic data from doubanjiang samples fermented by ICF and TOF. (**A**) Score plot of PCA; (**B**) Permutation test of the OPLS-DA model (based on 200 simulations). (**C**) Score plot of the OPLS-da model; (**D**) VIP scores of metabolites. The red frame highlights the key differential metabolites.

To effectively illustrate and distinguish the differences in the non-volatile profiles among doubanjiang with ICF and TOF, a PCA analysis was performed based on metabolite concentrations in doubanjiang, as depicted in Figure 5A. The first principal components together explained 41.2% of the total variance of all samples. Indeed, a clear separation between the ICF and TOF samples was obtained. To further identify key non-volatile compounds, OPLS-DA models were utilized. OPLS-DA further confirmed the separation between TOF and ICF samples, with satisfactory model parameters (R^2^X = 0.405, R^2^Y = 0.978, Q^2^ = 0.912). The reliability of the OPLS-DA model was assessed using a test based on 200 permutations (Figure 5B). Both R^2^ and Q^2^ values showed *p* < 0.01, and the intercept of the permuted Q^2^ regression line was −0.029, whereas the original model exhibited a high Q^2^ value of 0.912. These results indicate that the model had a strong predictive power and that the discrimination obtained was not due to overfitting. Based on the ^1^H-NMR data, a total of 23 compounds with VIP values exceeding 1 were screened between ICF and TOF samples, including alcohols and polyols (3), amino acids, peptides, and analogs (10), organic acids and derivatives (4), nucleotides and nucleosides (3), and others (3). Metabolites with VIP > 1.0 in the OPLS-DA model and FDR-adjusted *p* < 0.05 were considered important contributors to separation between the groups. Sixteen non-volatile metabolites met the criteria of VIP > 1.0 and FDR-adjusted *p* < 0.05, were considered as key differential metabolites and are therefore highlighted in the red box of Figure 5D as significantly different between ICF and TOF.

### 3.4. Pathway Analysis

**Figure 6:**
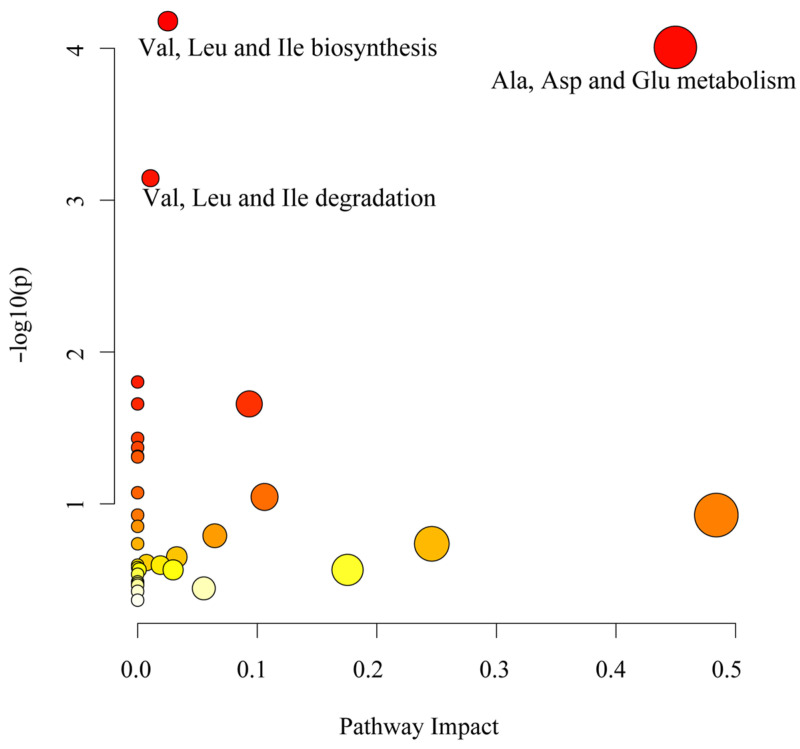
Pathway analysis for comparing the metabolic profiles of doubanjiang with ICF and TOF. The dimension of the dots underlines the pathway impact.

Pathway analysis was performed to elucidate the biochemical peculiarities underlying the metabolic differences between doubanjiang produced by ICF and traditional TOF (Figure 6). The differential metabolites identified from the two fermentation modes were mapped onto the KEGG pathways to assess pathway enrichment and pathway impact. The pathways with a *p*-value < 0.05 and path impact > 0 were considered significant.

In Figure 6, KEGG pathway analysis was performed to provide exploratory insights into potential metabolic pathways associated with the identified metabolites. In order of impact factor, these metabolic pathways include alanine, aspartate and glutamate metabolism, arginine biosynthesis, valine, leucine and isoleucine biosynthesis, and valine, leucine and isoleucine degradation. Notably, the alanine, aspartate and glutamate metabolism pathway together with the arginine biosynthesis pathway represented central nitrogen metabolism routes, suggesting that amino acid interconversion plays a key role in shaping the overall flavor matrix. Moreover, amino acid catabolism pathways may contribute to the formation of branched-chain aldehydes, alcohols, and acids detected in the GC-IMS analysis, which are important contributors to the overall volatile aroma profile.

### 3.5. Correlation Between Sensory Attributes and Key Differential Metabolites

**Figure 7:**
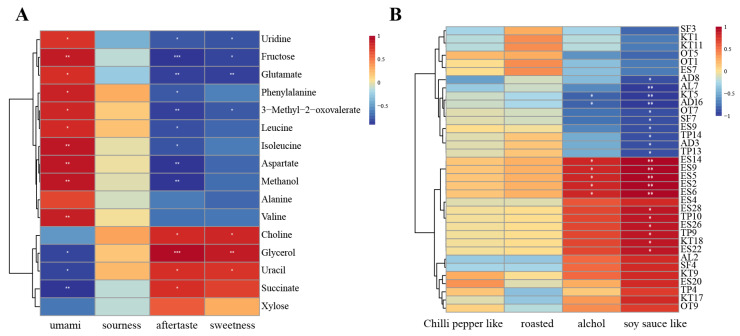
Correlation analysis between sensory attributes and key flavor compounds in PXDB. (**A**) Pearson correlation heatmap between sensory descriptors and key differential non-volatile metabolites identified by ^1^H-NMR. (**B**) Pearson correlation heatmap between sensory descriptors and key differential volatile compounds identified by GC-IMS. Asterisks indicate significant correlations (* *p* < 0.05, ** *p* < 0.01, *** *p* < 0.001).

To further explore the chemical basis underlying sensory perception differences between TOF and ICF, correlation analysis was performed between sensory attributes and the key differential non-volatile metabolites identified by ^1^H-NMR, as well as the key volatile compounds screened by GC-IMS (Figure 7).

The correlation heatmap between sensory descriptors and non-volatile metabolites (Figure 7A) revealed clear associations between taste attributes and key metabolites based on ^1^H-NMR analysis. Umami showed strong positive correlations with glutamate, aspartate, and arginine, highlighting the contribution of amino acid metabolism to savory taste formation. Sweet perception was positively correlated with fructose and ribose, reflecting the role of residual carbohydrates in modulating taste balance. The correlation analysis between sensory attributes and volatile compounds (Figure 7B) further demonstrated that ester compounds, particularly ethyl esters, were positively associated with soy sauce-like and alcohol aromas. In addition, branched-chain aldehydes and ketones exhibited positive associations with roasted aroma, suggesting their important contribution to the characteristic thermal-like notes in PXDB.

## 4. Discussion

PXDB, a traditional fermented condiment renowned for its unique flavor, exhibits strong regional specificity shaped by a complex matrix of volatile aroma compounds and non-volatile taste-active substances such as organic acids and free amino acids [15,16]. With the scale-up of industrial production, the manufacturing of PXDB has gradually shifted from TOF to ICF. However, multiple environmental factors, including temperature, oxygen availability, light exposure, and moisture content, significantly influence the quality attributes of PXDB, particularly its volatile and taste profiles [2,3]. These parameters are markedly altered by the transition from open to closed fermentation, leading to distinct differences in the flavor profiles between the two processes. In this study, the aroma and taste characteristics of PXDB produced by TOF and ICF were comprehensively compared through an integrative multi-analytical approach combining GC–MS for volatile profiling, ^1^H-NMR metabolomics, and sensory evaluation. Our results showed that ICF exhibited more pronounced soy sauce-like aroma and umami characteristics than TOF. This sensory divergence aligns with previous research on doubanjiang-meju fermentation, which similarly noted that controlled anaerobic conditions in closed systems favor the accumulation of umami-active amino acids and peptides [4]. Furthermore, the fermentation modality is associated with significant differences in both volatile and non-volatile metabolite compositions, leading to variations in flavor development and sensory perception between TOF and ICF.

Volatile compounds are the primary determinants of aroma perception in PXDB, and the present study clearly demonstrates that the fermentation mode may influence the volatile flavor formation of PXDB. GC-IMS analyses revealed pronounced differences between TOF and ICF, which may be associated with differences in oxygen availability and fermentation conditions. These differences suggest that fermentation mode may influence aroma profiles through changes in underlying metabolic processes. Regarding the discriminant variables, although 87 compounds displayed a VIP value > 1, the absence of variables with VIP > 1.2 suggests that the metabolic distinction between TOF and ICF is not governed by a single dominant marker but rather by the subtle, synergistic coordination of numerous moderate-impact metabolites. In addition, it should be noted that GC-IMS provides high sensitivity and rapid profiling of volatile compounds, but its compound identification relies primarily on database matching and lacks the structural confirmation capability of GC-MS. Therefore, the identified compounds in this study should be interpreted as tentative, and further validation using complementary analytical techniques is required.

Esters are key aroma-active compounds, contributing prominently to fruity, sweet, and sauce-like sensory attributes. In this study, the total relative proportion of esters was significantly higher in ICF than in TOF, with particular reference to ethyl esters. The significantly higher proportion of esters in ICF indicates that the hermetic environment provides a more favorable metabolic landscape for esterification. In the closed system, the lower oxidation rate of alcohols—compared to the constant atmospheric exposure in TOF—leads to a higher concentration of ethanol, which serves as a critical substrate for the synthesis of ethyl esters. This suggests that ester formation in PXDB is strongly influenced by controlled fermentation conditions, rather than fermentation openness alone. In fact, ester concentrations were also found to be increased in PXDB fermented in a closed system, when gradients of temperature were applied [2,17]. Moreover, sensory evaluation revealed that ICF exhibited a significantly higher intensity of soy sauce-like aroma. Ethyl esters are mainly formed via esterification between ethanol and medium-chain fatty acids, a pathway closely linked to yeast metabolism and intracellular acyl-CoA availability [18,19]. The higher ethyl ester levels in ICF may be related to its strictly controlled anaerobic and thermally stabilized fermentation environment, whereas environmental variability in TOF may promote ester hydrolysis or volatilization. Notably, methyl 2-methylbutyrate, isoamyl acetate, and ethyl hexanoate exhibited more than 2-fold higher relative peak areas in ICF samples. These compounds have been previously confirmed as key contributors to the characteristic fruity and fermented aroma of PXDB [20,21]. Furthermore, the stable temperature profiles in ICF facilitate the activity of alcohol acyltransferases, which are the primary enzymes responsible for ester synthesis during fermentation. In contrast, the open-air exposure in TOF likely facilitates the oxidation of ethanol into acetic acid, thereby depleting the precursor pool required for ester formation and potentially shifting the metabolic trajectory toward the production of higher alcohols and organic acids [17].

Alcohols that exhibit pleasant floral aroma and fruit sweetness act as key metabolic hubs linking amino acid catabolism with downstream aroma formation, including aldehydes and esters [22,23]. Higher alcohols, typically derived from amino acid catabolism via the Ehrlich pathway, were more abundant in TOF, which may reflect differences in amino acid metabolism under different fermentation conditions [24,25,26]. Among them, 1-butanol and 3-methyl-1-butanol deserve particular attention, as both have been reported as key aroma-active compounds in PXDB [20,21]. 3-Methyl-1-butanol, described as having whiskey, banana, and fruity-like notes [27], is typically produced via the Ehrlich pathway from leucine or isoleucine and is commonly associated with microbial metabolism [28]. Its markedly higher relative abundance in TOF suggests that the more diverse and dynamic microbial environment in open fermentation enhances branched-chain amino acid catabolism [6]. In contrast, 1-butanol, characterized by a wine-like aroma, exhibited a relative concentration more than two-fold higher in ICF than in TOF. The opposite trends observed for these two key alcohols highlight that fermentation mode not only affects the total alcohol content but also reshapes the balance between structurally and sensorially distinct alcohols, thereby contributing to the divergent aroma profiles of ICF and TOF.

Ketones showed a significantly higher overall relative presence in ICF than in TOF, suggesting that closed fermentation appears to favor ketone accumulation in PXDB. However, the distribution of individual ketones differed between the two fermentation modes. Among the screened differential compounds, 2-heptanone-M, 1-hydroxy-2-propanone, and 4-methyl-3-penten-2-one exhibited significant higher (FDR-adjusted *p* < 0.05, and fold change > 2) relative peak areas in TOF, whereas 4-hexen-3-one was more abundant in ICF. This suggests that although ICF generally promotes ketone formation, TOF favors the accumulation of specific ketones, which may be associated with differences in oxidation conditions and fermentation environment [2]. The contrasting patterns further indicate that fermentation mode may influence ketone profiles through different metabolic processes, contributing to subtle but meaningful differences in the aroma characteristics of ICF and TOF.

Aldehydes, acids, terpenes, sulfur compounds and furans are other classes of compounds with known effects on the aroma of PXDB [16,29]. However, the relative proportion of aldehydes did not differ significantly between the two fermentation modes, suggesting that their formation was not markedly affected by the fermentation strategy. Similarly, although acids exhibited a higher overall relative proportion in ICF, none of the acids quantified met the combined screening criteria of VIP value, FDR-adjusted *p* < 0.05, and fold change >2 or <0.5. These results indicate that aldehydes and acids remained relatively stable across fermentation modes and were not the primary drivers of aroma discrimination between ICF and TOF, in contrast to esters and alcohols. Terpenes exhibited a significantly higher relative proportion in TOF than in ICF, mainly driven by linalool oxide, whose relative peak area in TOF was approximately seven times higher than ICF, while linalool itself did not differ significantly between the two fermentation modes. This suggests that TOF may promote oxidative or biotransformation processes converting linalool into its oxygenated derivatives during the chili fermentation, rather than increasing terpene precursors [16]. In addition, sulfur compounds were markedly enriched in TOF, accounting for 8.3% of total volatiles compared with 2.3% in ICF. Diallyl sulfide, characterized by a garlic-like odor, was a key differential compound enriched in TOF, whereas dimethyl trisulfide, associated with fresh onion, mint, and spicy notes, was identified as a characteristic sulfur compound in ICF [30]. These results indicate that open fermentation promotes the formation of terpene derivatives and sulfur compounds, contributing to the more pungent and complex aroma profile of TOF.

In addition to volatile compounds, non-volatile metabolites played an equally crucial role in shaping the taste attributes of PXDB. Among them, amino acids represent one of the most important groups of flavor-active substances, as they contribute directly to basic taste sensations and also serve as precursors for multiple aroma-generating pathways. In the present study, a total of 18 amino acids were identified in PXDB, many of which have been previously reported as contributors to umami, sweet, or bitter taste [31,32]. Seven amino acids, including glutamate, aspartate, alanine, isoleucine, leucine, valine, and phenylalanine, were screened as key discriminant metabolites distinguishing TOF from ICF. This pattern suggests that amino acid profiles are sensitive to fermentation conditions, and to the balance between proteolysis and amino acid catabolism [33]. Phenylalanine, significantly higher in TOF, is a precursor of phenylacetaldehyde, a compound that imparts honey-like or floral notes. Its higher concentration in TOF aligns with the GC–IMS results, showing richer aromatic complexity in TOF samples. Conversely, the other six differential amino acids were significantly higher in ICF. Glutamate, the amino acid mainly responsible for umami taste, showed the highest concentration in both types of PXDB, consistently with earlier findings in PXDB products [4,22,32]. Its abundance is likely attributable to extensive protein degradation by microbial proteases derived from Aspergillus, yeasts, and bacteria involved in meju fermentation [19]. Aspartate, another amino acid strongly contributing to umami taste [32], also exhibited significantly higher levels in ICF. The elevated concentrations of both glutamate and aspartate indicate that proteolysis under closed fermentation conditions may be more efficient for releasing acidic amino acids under closed fermentation. The results indicate that the ICF samples had a more umami-oriented amino acid profile than those of the TOF. As confirmation, the significant positive correlations between umami perception and amino acid-related metabolites such as glutamate, aspartate, and arginine suggest that nitrogen metabolism is a major determinant of taste quality in PXDB. This finding is consistent with the pathway enrichment results, where alanine, aspartate, glutamate metabolism and arginine biosynthesis were identified as the most impacted pathways.

Organic acids exhibited substantial variation between the two fermentation modes. Lactate, acetate, and succinate were the major organic acids in our samples. Succinate, an intermediate of the TCA cycle, was more abundant in TOF, which may be associated with more active oxidative processes. Its presence contributes not only to sourness but also to the overall body and mouthfeel of the product. In contrast, 3-methyl-2-oxovalerate, an α-keto acid produced from isoleucine via branched-chain amino acid transamination, was more highly accumulated in ICF, consistent with the higher levels of isoleucine observed in ICF. This metabolite serves as an important precursor for branched-chain aldehydes, alcohols, and acids that contribute fruity, malty, and roasted notes to the final flavor profile [23,33]. These changes correspond closely with the sensory evaluation results, which showed stronger sourness in ICF.

Overall carbohydrate concentration showed a direct correlation with the sensory analysis of PXDB [19]. In detail, seven sugars were detected by ^1^H-NMR, including arabinose, fructose, fucose, galactose, glucose, ribose, and xylose. Among them, fructose showed the highest levels in ICF samples. In contrast, carbohydrate levels were more rapidly depleted in TOF, likely due to enhanced metabolic activity under open fermentation conditions [32]. The higher retention of residual sugars in ICF may contribute to sweetness perception, whereas the accelerated sugar consumption in TOF provides substrates for the formation of volatile compounds, linking non-volatile and volatile metabolic pathways. The interplay between sugar degradation, amino acid catabolism, and ester synthesis underscores the metabolic integration that contributes to the rich aroma and taste complexity in TOF.

Collectively, these observations indicate that TOF benefits from a dynamic ecological environment in which the open fermentation environment may favor more diverse metabolic activities [2,4]. In contrast, the more controlled ICF environment promotes consistency but may limit certain metabolic processes, thereby limiting aromatic diversity. These findings may provide insights for optimizing ICF by targeting introduction of controlled oxygen exposure, co-fermentation with selected yeasts, or modulation of fermentation temperature, which may enhance flavor complexity without compromising production efficiency. Understanding these metabolic and sensory differences not only provides scientific insight into flavor formation in PXDB but also offers a foundation for tailoring industrial fermentation strategies to approach the sensory richness of traditionally fermented products. From a metabolomic perspective, these differences reflect a shift from oxidation- and amino acid-driven flavor formation in TOF to an umami-oriented amino acid profile and an ester-oriented aroma profile in ICF. It should be noted that the observed differences are based on samples from a single production facility, and further studies including multiple manufacturers are required to validate the generality of these findings. In this context, the role of the starter culture, initially standardized, becomes central when viewed through the lens of microbial community succession. Furthermore, while a rigorous double-blind protocol, randomized coding, and standardized training with objective flavor references were employed to maximize objectivity, the affiliation of the panelists with the production company remains a potential limitation. The panelists’ inherent familiarity with the manufacturer’s typical product profile could shape their sensory thresholds or descriptive evaluations, representing a potential source of bias. This factor should be taken into consideration when interpreting the sensory results. Future studies integrating microbiological analyses are needed to further elucidate how different fermentation setups influence microbial community dynamics and metabolic pathways underlying flavor formation in PXDB.

## 5. Conclusions

This study provides a controlled comparison of TOF and ICF, demonstrating that fermentation setup is a key factor shaping both volatile and non-volatile flavor profiles in PXDB. By integrating GC-IMS, ^1^H-NMR metabolomics, and sensory evaluation, this study provides a systematic comparison of traditional open and industrial closed fermentation systems and establishes a comprehensive framework for linking fermentation conditions with flavor characteristics in PXDB. The results indicate that fermentation mode is likely associated with differences in both volatile aroma composition and non-volatile taste-active metabolites, contributing to perceptible differences in sensory attributes between TOF and ICF products. ICF was characterized by higher proportions of esters, ketones, and umami-related amino acids, particularly glutamate and aspartate, which contributed to a stronger soy sauce-like aroma and enhanced umami taste. In contrast, TOF promoted the accumulation of alcohols, terpene derivatives, sulfur compounds, and selected organic acids, resulting in a more complex and pungent aroma profile. These differences may reflect variations in metabolic processes potentially associated with fermentation environment, microbial activity, and substrate utilization under open versus closed processing conditions. Overall, the findings indicate that flavor formation in PXDB is governed by coordinated changes in amino acid metabolism, lipid-derived pathways, and carbohydrate transformation, rather than by individual compounds alone. While these observations provide insights into possible underlying mechanisms, further studies including microbiological and metabolic analyses are needed to directly validate these hypotheses in multiple production batches with different raw material sources. To improve product consistency while retaining traditional sensory complexity, we recommend that industrial producers consider diversifying fermentation environmental parameters to emulate the metabolic profile of TOF without compromising the safety and efficiency of the ICF. This study provides insights into optimizing industrial fermentation strategies to balance product consistency with traditional sensory complexity, while preserving the traditional characteristics of this iconic fermented food. However, these findings should be interpreted as exploratory and context-dependent, and further studies with independent biological replicates across multiple production systems are required to validate these observations.

## Data Availability

The original contributions presented in this study are included in the article/Supplementary Materials. Further inquiries can be directed to the corresponding author.

## Supplementary Materials

The following supporting information can be downloaded at: https://www.mdpi.com/article/10.3390/foods15132384/s1, Figure S1: Comparative schematic illustration of the fermentation process and sampling strategy of PXDB under traditional open fermentation (TOF) and industrial closed fermentation (ICF); Figure S2: Portions of a representative ^1^H-NMR spectrum of Doubanjiang, selected to evidence, for each molecule quantified, the signal employed for its quantification. The vertical zoom of each portion has been selected to ease its visual inspection; Figure S3: Pictorial representation of the global spectral deconvolution procedure, implemented in MestReNova. Its effectiveness can be visually evaluated (A) in a case of severe superimposion of signals and (B) in the case of a particularly crowded region; Figures S4–S17: Pictorial description of the molecules’ assignment procedure by Chenomx software (ver 8.5). Upper panel—portions of the representative spectrum. Lower panel—The spectrum (black line) superimposed to the simulated signals (red line) for each of the molecules listed. The line connecting each name with the spectra highlights the signal used for quantification purposes; Table S1: QDA sensory values (Mean ± SD) of Pixian Doubanjiang produced by traditional open fermentation (TOF) and industrial close fermentation (ICF); Table S2: Peak areas of volatile compounds determined by GC-IMS in the PXDB with traditional open fermentation (TOF) and industrial close fermentation (ICF); Table S3: VIP values of OPLS-da based on the GC-IMS data; Table S4: Concentrations (mmol/g, mean ± sd) of molecules determined by ^1^H-NMR in the PXDB with traditional open fermentation (TOF) and industrial close fermentation (ICF).
