# Supplementary material for: Comparison of Aroma and Taste Profiles of Pixian Douban Fermented with Traditional Open Process or Industrial Closed Process
Source: Foods. 2026 Jul 3;15(13):2384. doi: 10.3390/foods15132384 (PMC13361808; doi:10.3390/foods15132384)
Supplement: Supplementary file 1 [file foods-15-02384-s001.zip › foods-4363494-supplementary.pdf]

Comparison of Aroma and Taste Profiles of Pixian Douban Fermented with Traditional Open Process or Industrial Closed Process

Supplemental material

Figure S1. Comparative schematic illustration of the fermentation process and sampling strategy of PXDB under traditional open fermentation (TOF) and industrial closed fermentation (ICF)

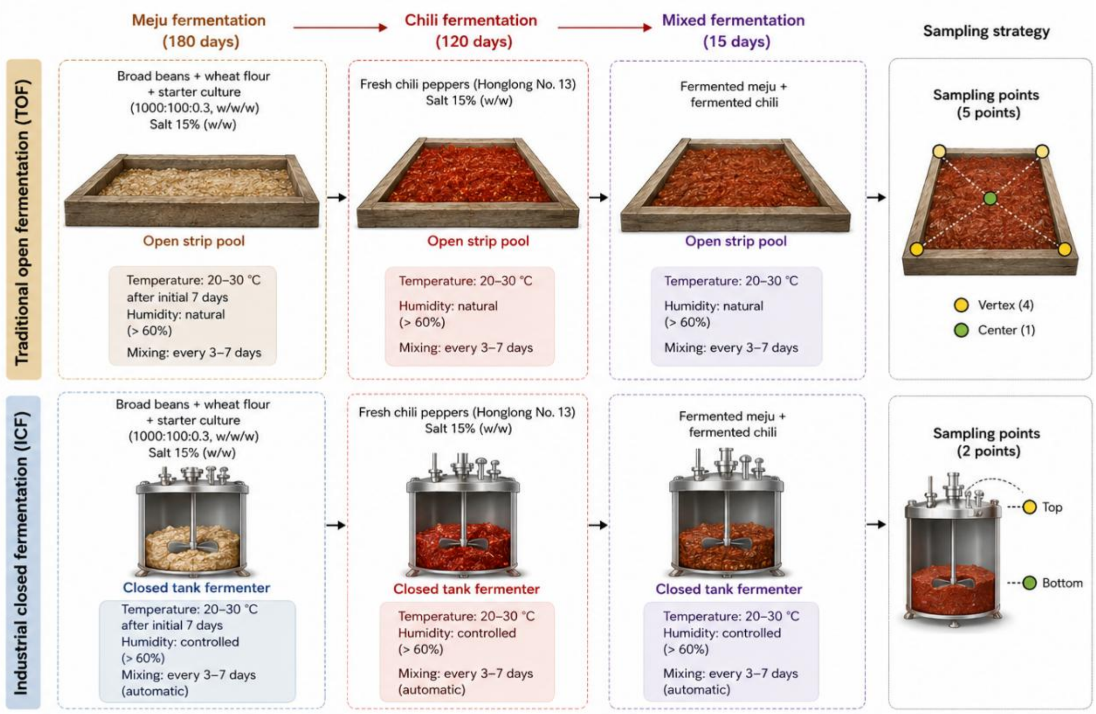

Figure S2. Portions of a representative ^{1}H-NMR spectrum of Doubanjiang, selected to evidence, for each molecule quantified, the signal employed for its quantification. The vertical zoom of each portion has been selected to ease its visual inspection.

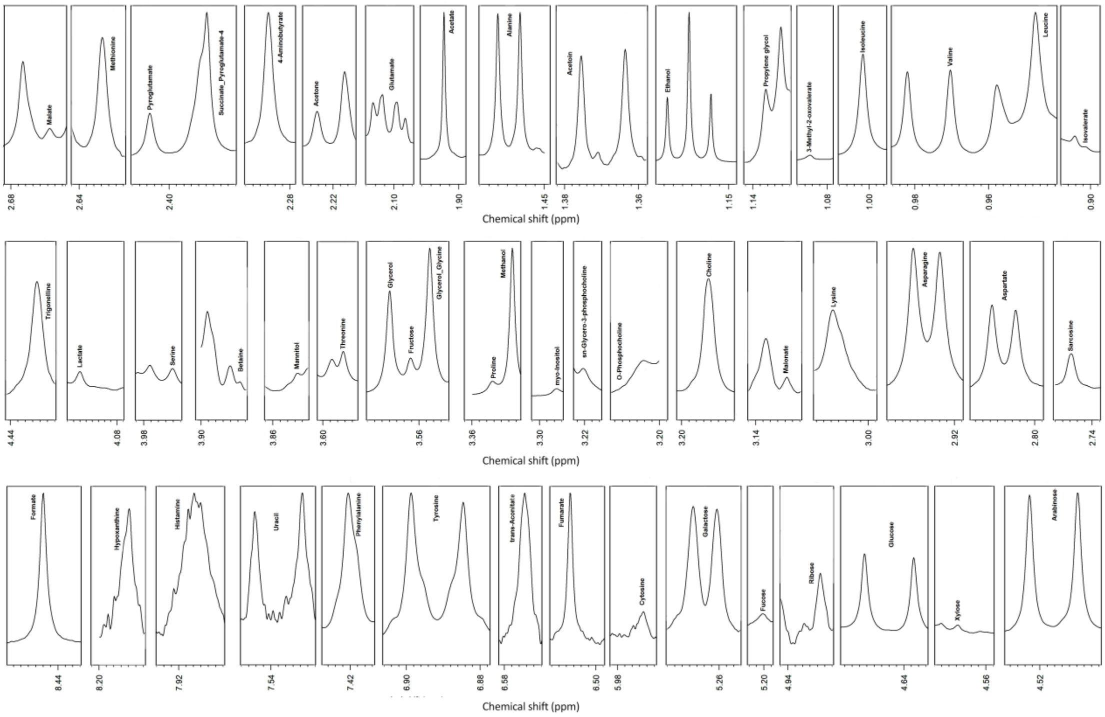

Figure S3: Pictorial representation of the global spectral deconvolution procedure, implemented in MestReNova. Its effectiveness can be visually evaluated A) in a case of severe superimposion of signals and B) in the case of a particularly crowded region.

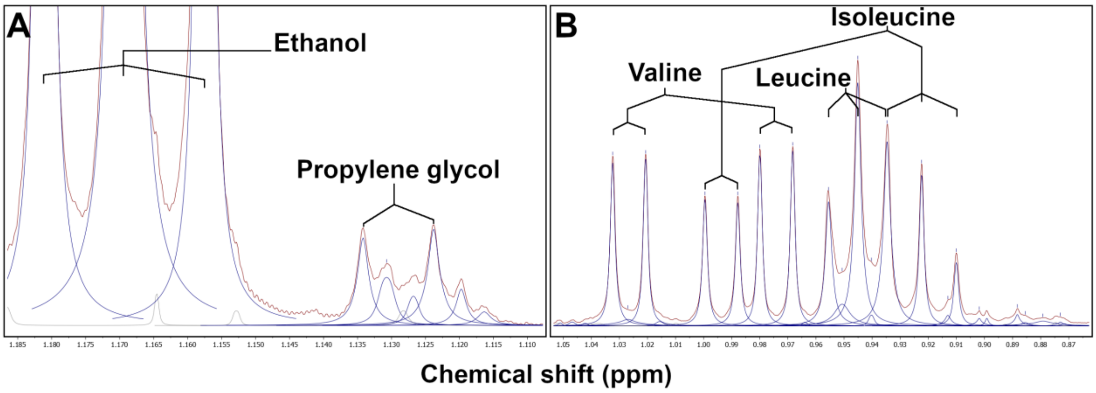

Figures S4-S17. Pictorial description of the molecules' assignment procedure by Chenomx software. Upper panel - portions of the representative spectrum. Lower panel - The spectrum (black line) superimposed to the simulated signals (red line) for each of the molecules listed. The line connecting each name with the spectra highlights the signal used for quantification purposes.

Figure S4

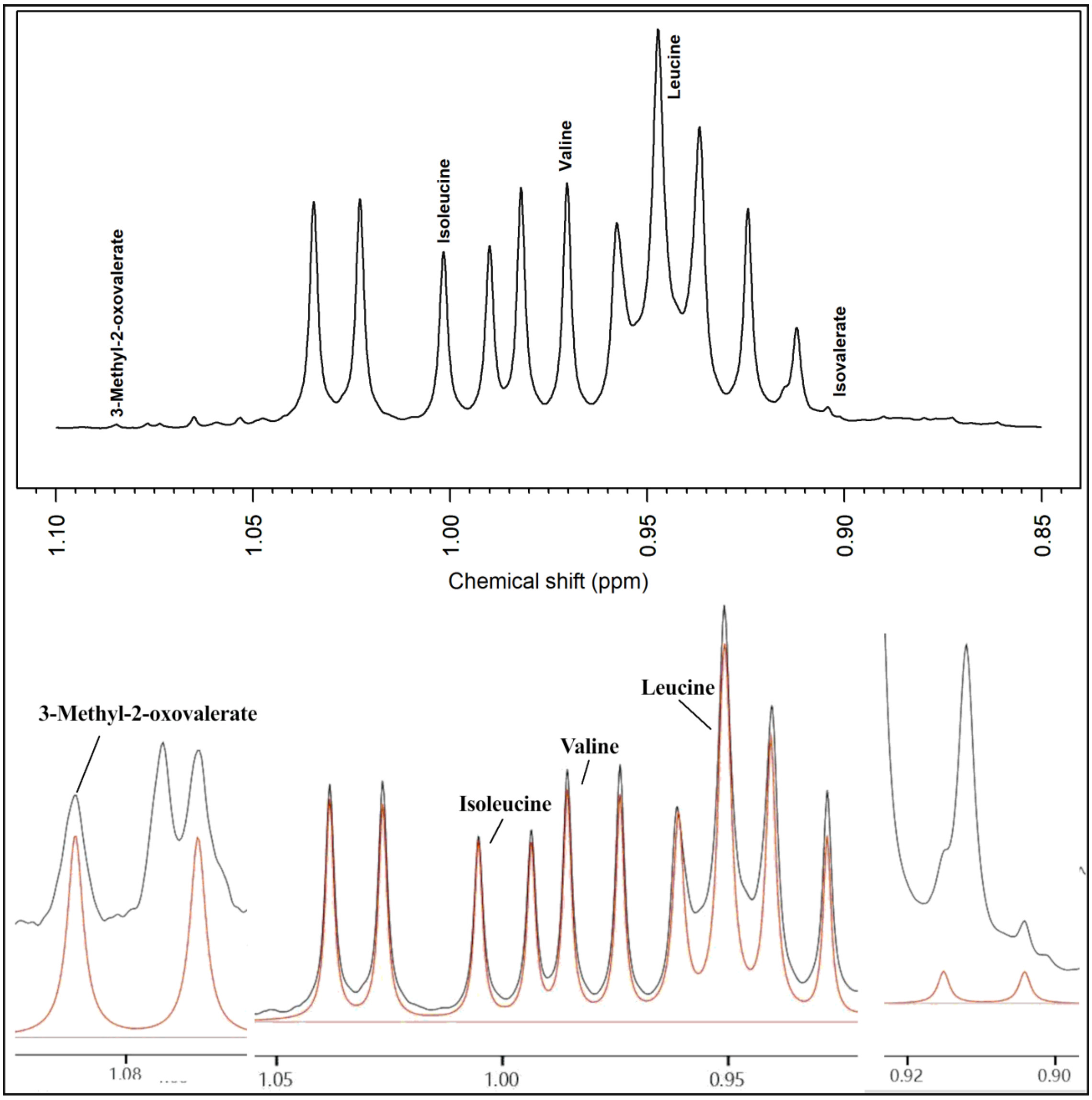

Figure S5

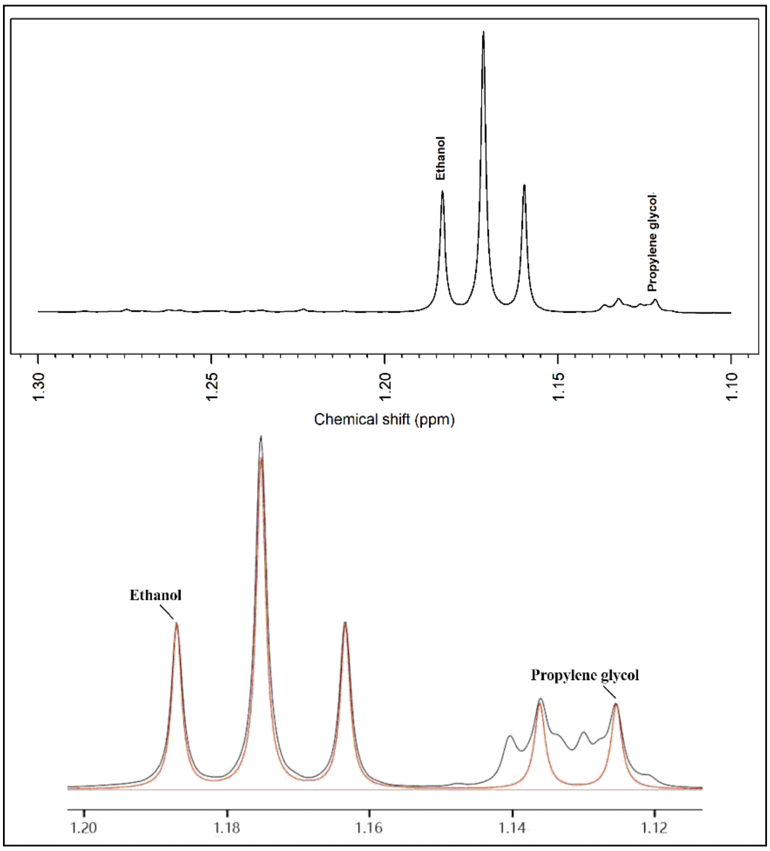

Figure S6

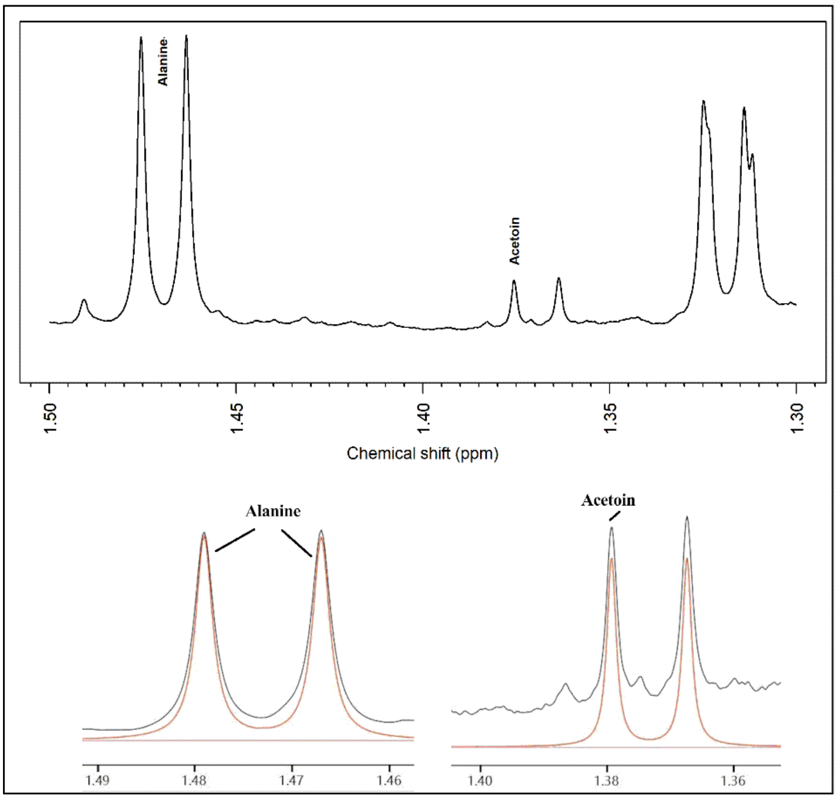

Figure S7

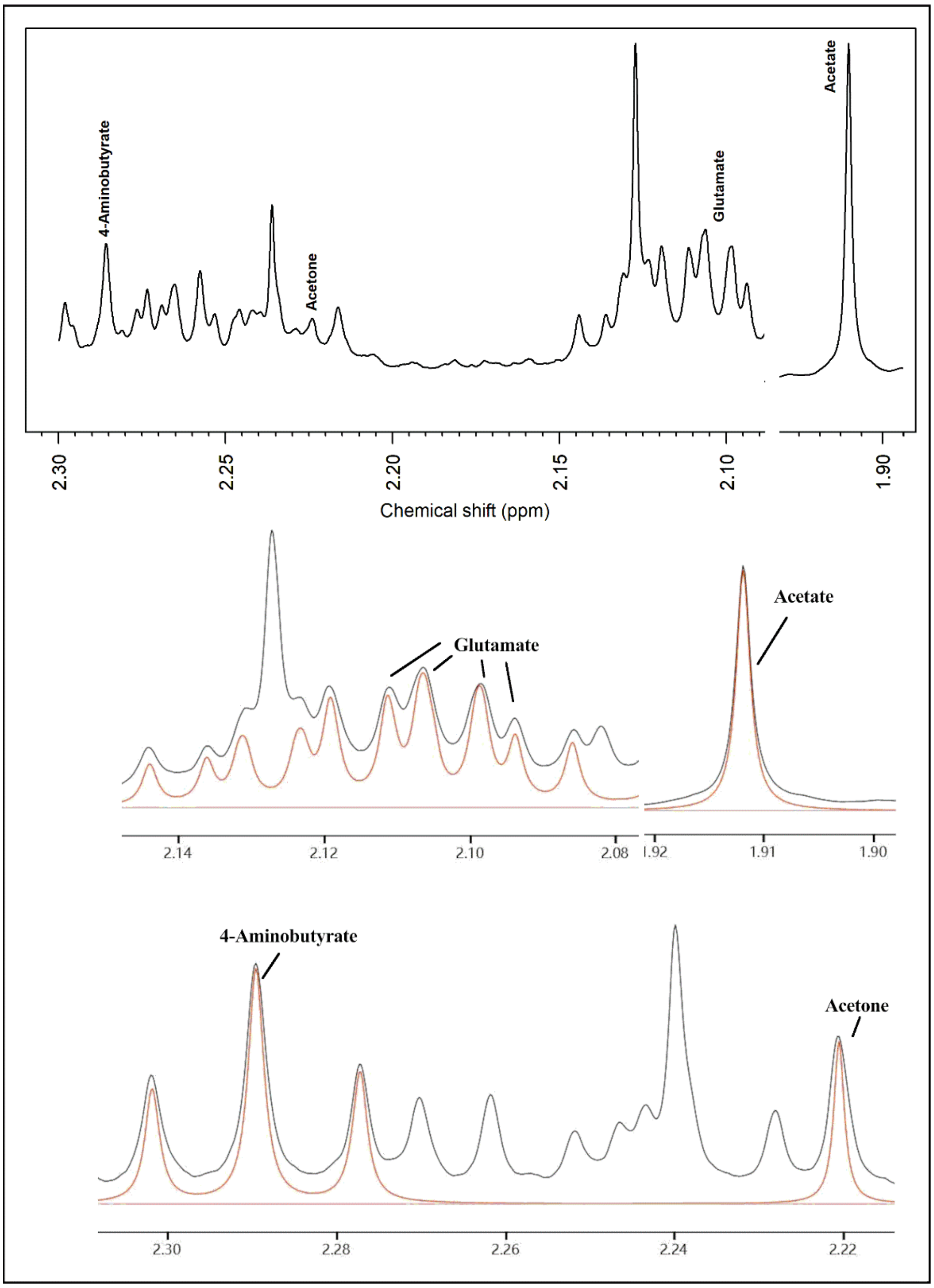
4-Aminobutyrate
Acetone
Glutamate
Acetate
2.30
2.25
2.20
2.15
2.10
1.90
Chemical shift (ppm)
Glutamate
Acetate
2.14
2.12
2.10
2.08
1.92
1.91
1.90
4-Aminobutyrate
Acetone
2.30
2.28
2.26
2.24
2.22

Figure S8

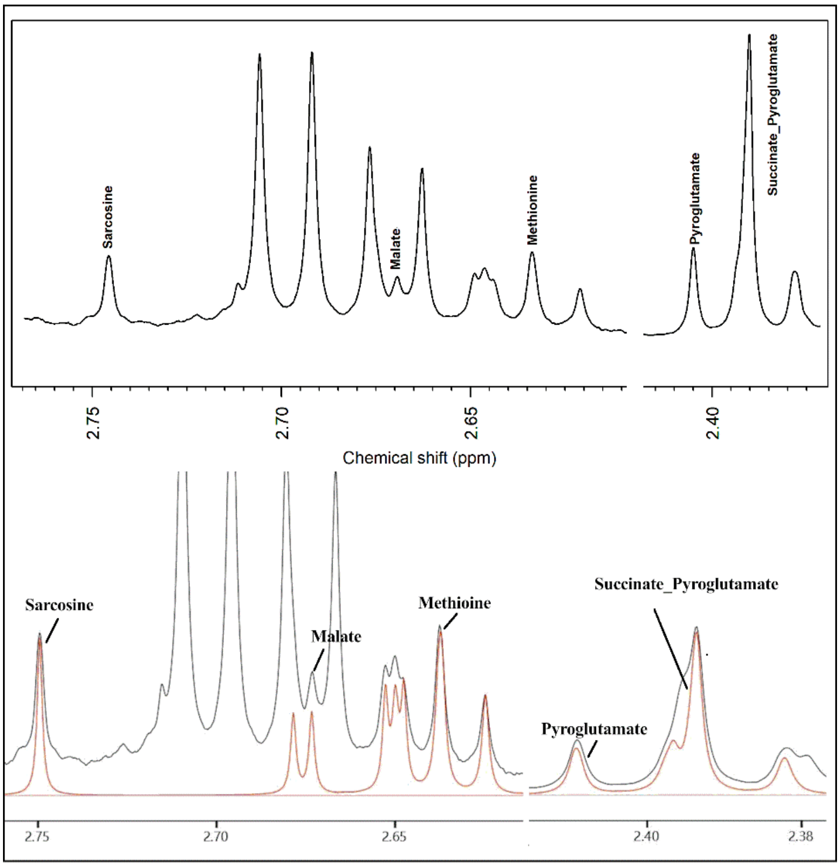

Figure S9

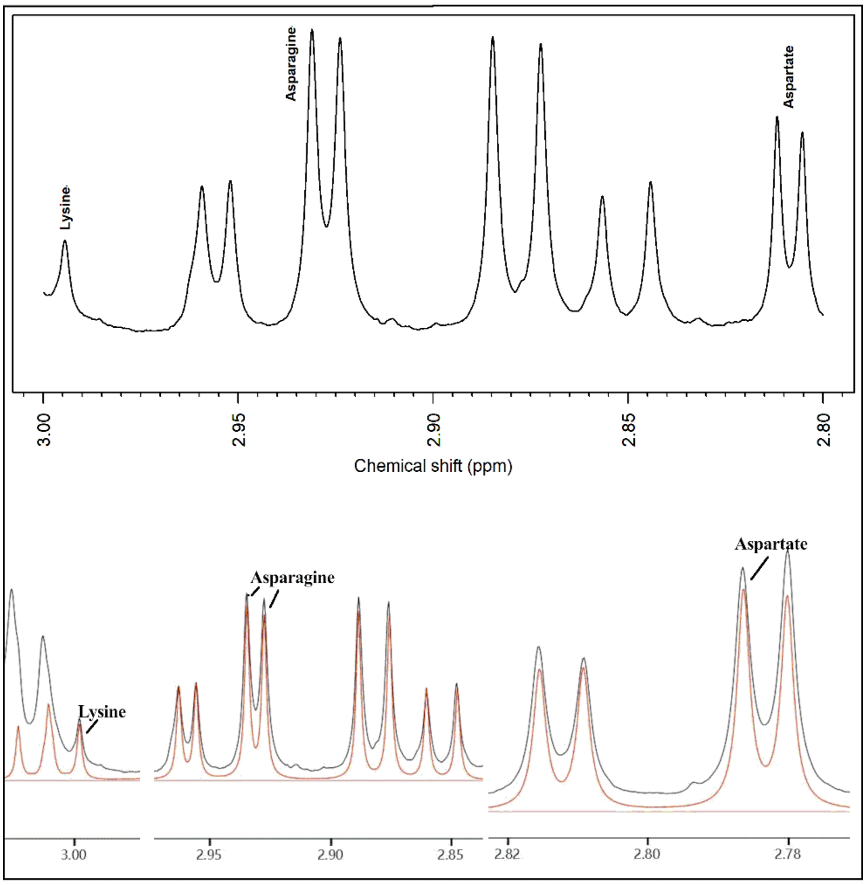

Figure S10

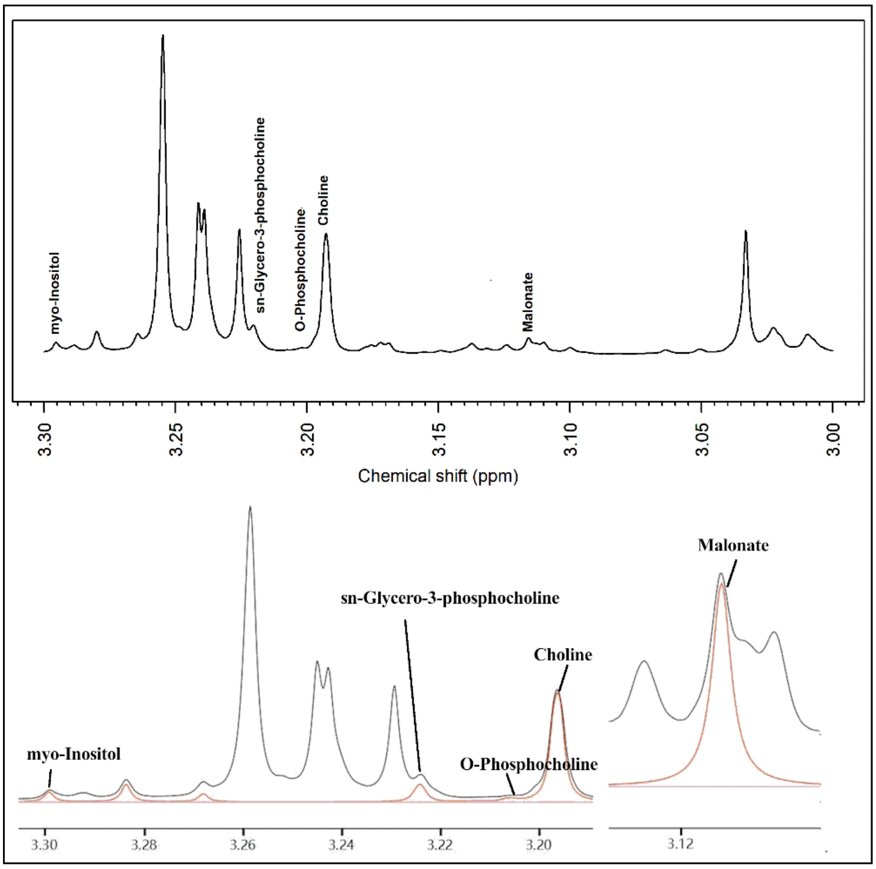

Figure S11

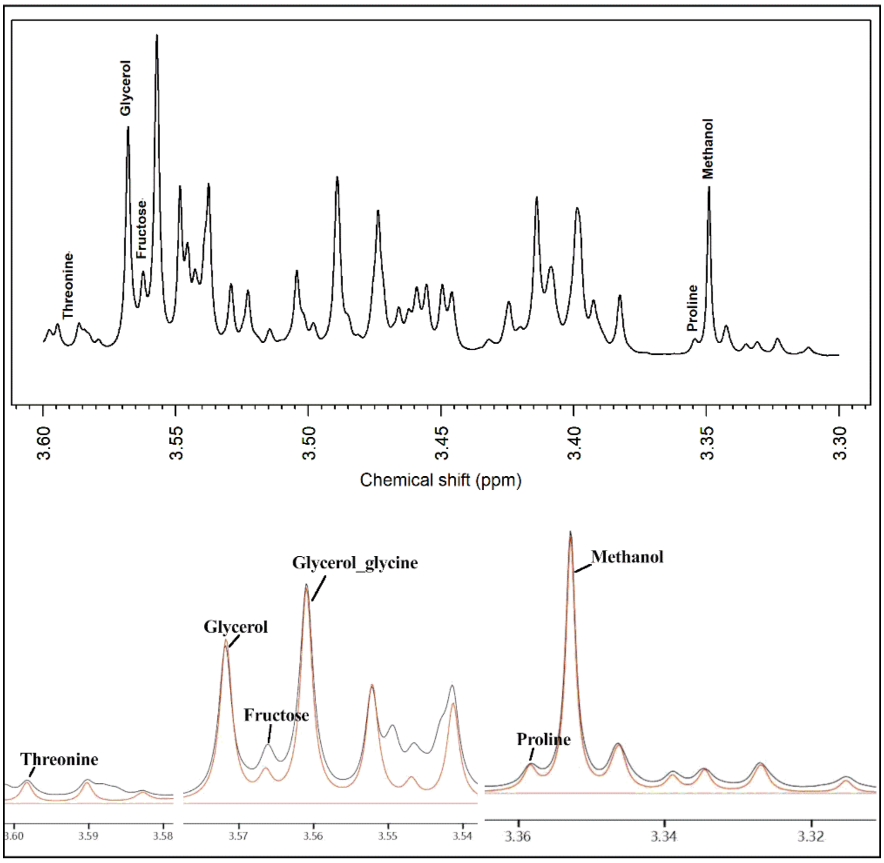

Figure S12

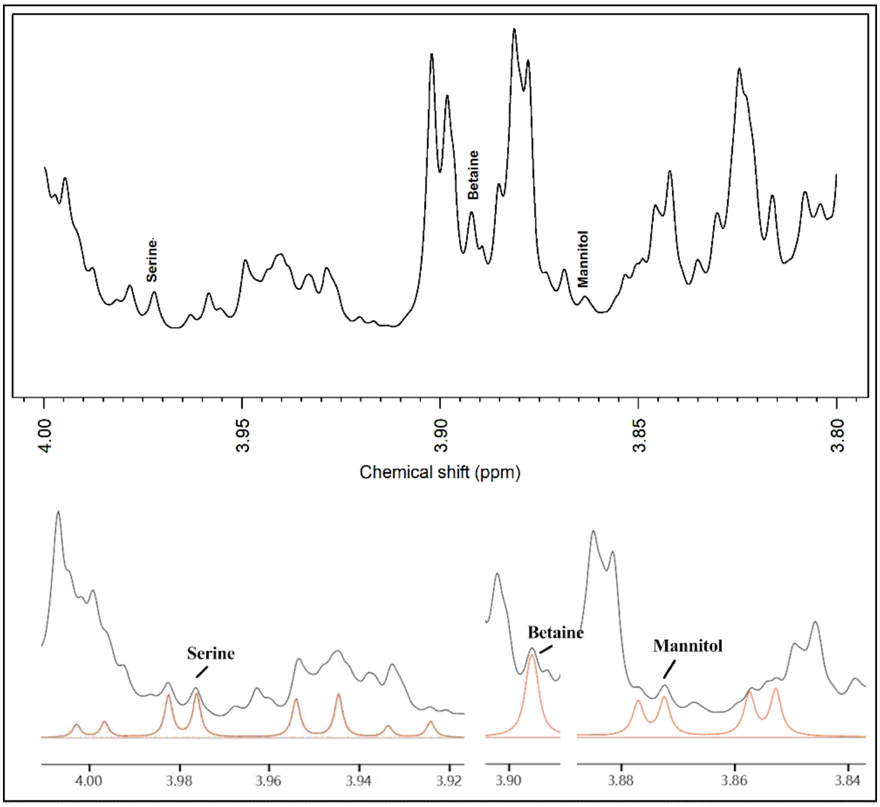

Figure S13

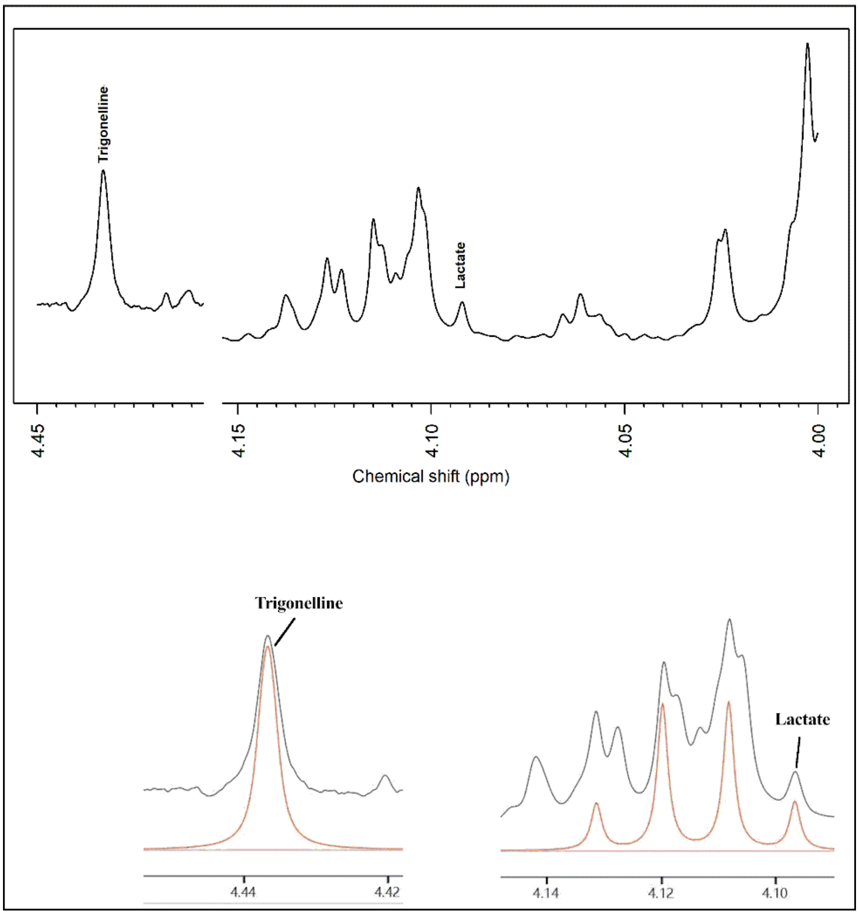

Figure S14

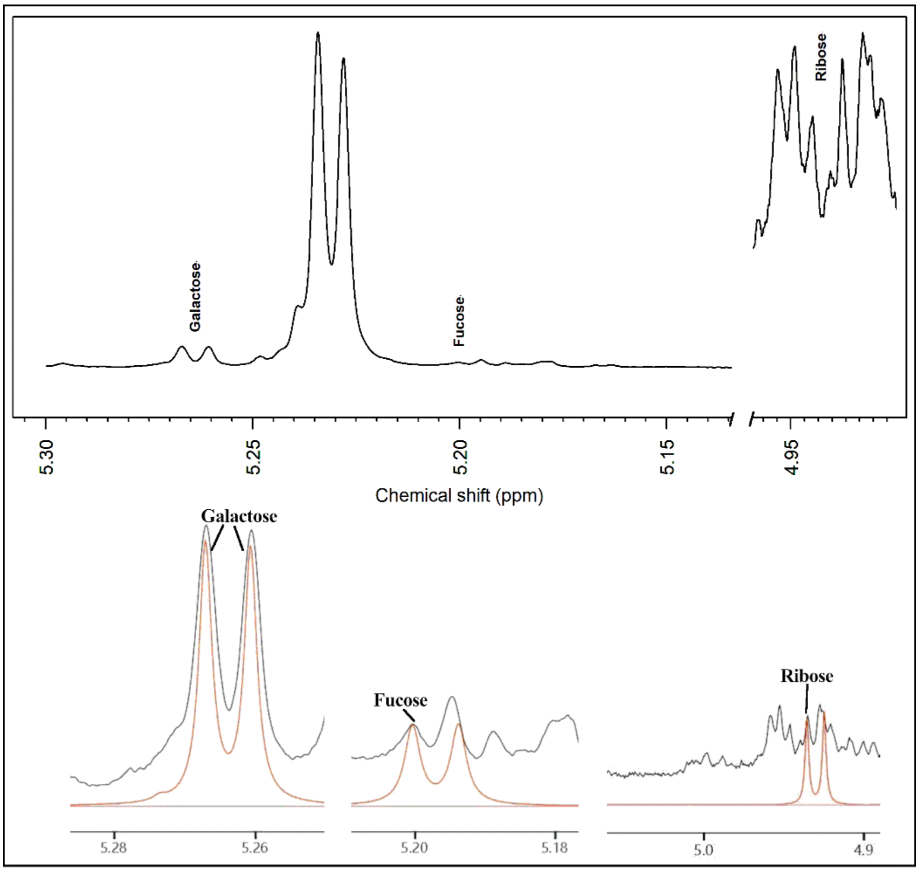

Figure S15

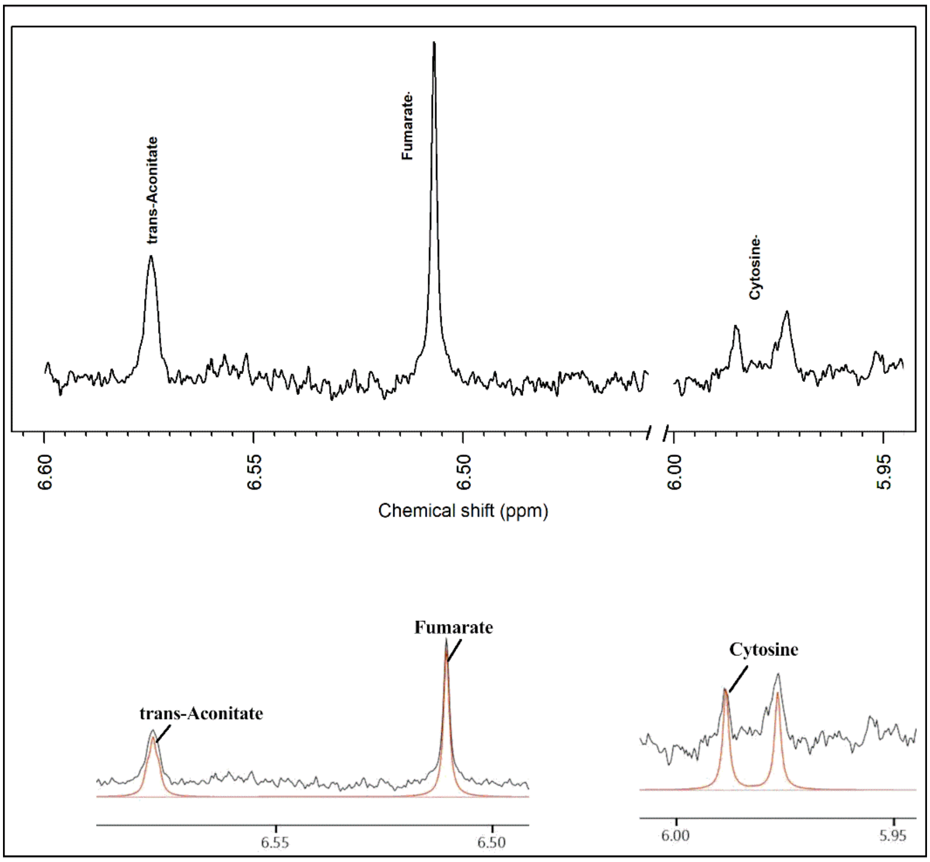

Figure S16

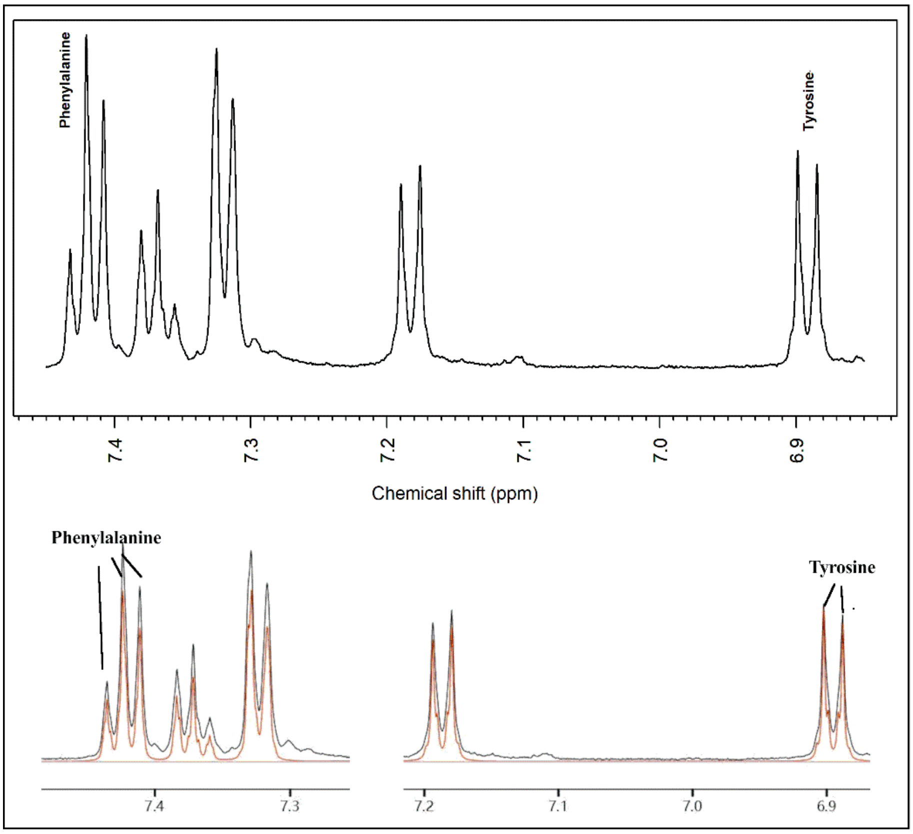

Figure S17

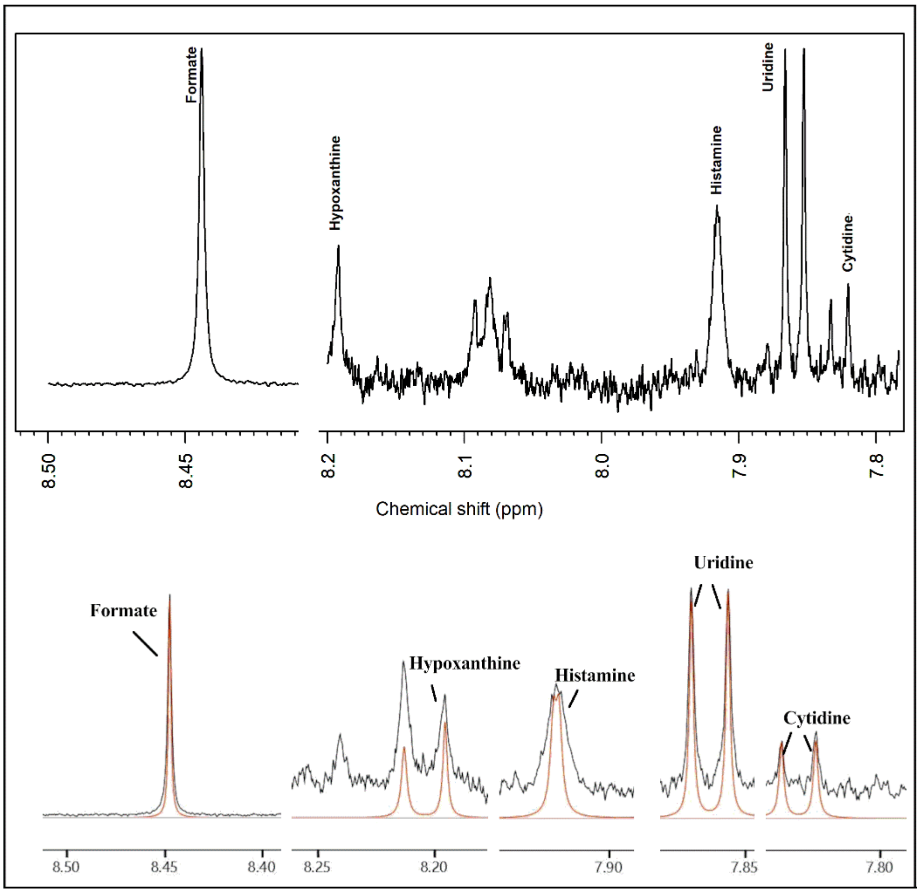

Table S1. QDA sensory values (Mean±SD) of Pixian Doubanjiang produced by traditional open fermentation (TOF) and industrial close fermentation (ICF).

	ICF (Mean±SD)	TOF (Mean±SD)	P-value	FDR	Significant difference (ICF vs TOF)
Soy sauce	6.60±0.52	5.20±1.03	0.0015	0.0135	↗*
Chilli pepper-like	4.90±1.97	4.60±1.17	0.7	0.84	ns
Alchol-like	5.20±0.79	4.40±0.84	0.048	0.216	ns
Roasted	2.80±0.92	3.10±1.10	0.494	0.658	ns
Umami	5.80±0.79	4.70±0.48	0.0028	0.0168	↗
Sourness	3.30±0.67	3.70±0.67	0.175	0.35	ns
Sweetness	2.80±0.63	3.50±0.71	0.022	0.099	ns
Aftertaste	4.80±0.63	5.60±1.17	0.067	0.067	↘

* ↗ and ↘ indicate significantly higher or lower relative contents in ICF compared with TOF (FDR-adjusted $p < 0.05$), respectively

# ns indicates no significant difference.

P-values were adjusted using the Benjamini-Hochberg FDR correction.

Table S2. Peak areas of volatile compounds determined by GC-IMS in the PXDB with traditional open fermentation (TOF) and industrial close fermentation (ICF).

Compound	Label	CAS	Aroma description	TOF	ICF	ICF vs TOF
Esters						
2-Methylbutyl acetate	ES1	624-41-9	fruity	$5.55\times10^{2}\pm7.24$	$6.01\times10^{2}\pm9.77\times10$	ns[#]
2-Methylpropyl acetate	ES2	110-19-0	fruity, raw pear and raspberry	$3.75\times10^{2}\pm1.08\times10$	$9.77\times10^{2}\pm6.46\times10$	↗[*]
3-Methyl-2(5H)-furanone	ES3	22122-36-7	cooked, roasted	$8.44\times10^{2}\pm1.11\times10^{2}$	$9.90\times10^{2}\pm3.45\times10$	ns
3-Methylbutyl propanoate	ES4	105-68-0	fruity	$2.53\times10\pm3.70$	$9.74\times10\pm8.82$	↗
Ethyl (E)-2-butenoate	ES5	623-70-1	sourness, fruity, rum ether	$2.41\times10^{2}\pm2.38\times10$	$2.38\times10^{3}\pm2.05\times10^{2}$	↘
Ethyl 2-methylbutanoate	ES6	7452-79-1	apple	$2.69\times10^{3}\pm9.96\times10$	$5.81\times10^{3}\pm2.49\times10^{2}$	↗
Ethyl 3-hydroxybutanoate	ES7	5405-41-4	fruit, grape, green and wine	$2.55\times10^{2}\pm1.36\times10$	$5.33\times10\pm4.45$	↘
Ethyl 3-methylbutanoate-D	ES8	108-64-5	apple, banana, sour and sweet	$3.67\times10^{3}\pm1.24\times10^{2}$	$7.71\times10^{3}\pm3.23\times10^{2}$	↗
Ethyl 3-methylbutanoate-M	ES9	108-64-5	apple, banana, sour and sweet	$1.78\times10^{3}\pm4.07\times10$	$8.58\times10^{2}\pm9.85$	↘
Ethyl acetate	ES10	141-78-6	fresh, fruity, sweet, grassy	$8.26\times10^{3}\pm7.90\times10$	$9.37\times10^{3}\pm5.38\times10$	↗
Ethyl benzoate	ES11	93-89-0	camomile, floral, celery, fruity	$5.13\times10^{2}\pm2.20\times10$	$6.31\times10^{2}\pm2.45\times10$	↗
Ethyl butanoate	ES12	105-54-4	pineapple, fruity, ester, whiskey	$4.38\times10^{3}\pm5.06\times10$	$5.67\times10^{3}\pm2.22\times10^{2}$	↗
Ethyl decanoate	ES13	110-38-3	fruity, wine, pear, brandy	$2.55\times10^{2}\pm4.90\times10$	$4.05\times10^{2}\pm3.41\times10$	↗
Ethyl hexanoate-D	ES14	123-66-0	pineapple, fruity, wine	$3.00\times10^{2}\pm1.88\times10$	$1.36\times10^{3}\pm1.00\times10^{2}$	↗
Ethyl hexanoate-M	ES15	123-66-0	pineapple, fruity, wine	$1.14\times10^{3}\pm4.34\times10$	$2.02\times10^{3}\pm4.51\times10$	↗
Ethyl isobutyrate	ES16	97-62-1	sweet, fruity, alcoholic, rummy	$3.64\times10^{3}\pm5.43\times10$	$5.88\times10^{3}\pm8.75\times10$	↗
Ethyl lactate-D	ES17	97-64-3	fruity	$7.29\times10^{2}\pm4.15\times10$	$1.18\times10^{3}\pm5.50\times10$	↗
Ethyl lactate-M	ES18	97-64-3	fruity	$4.73\times10^{2}\pm3.32$	$7.00\times10^{2}\pm2.50\times10$	↗
Ethyl nonanoate	ES19	123-29-5	banana, grape, fruity, wine, rose, fatty, waxy	$1.78\times10^{2}\pm3.31\times10$	$1.90\times10^{2}\pm2.81$	ns
Ethyl octanoate-D	ES20	106-32-1	fruity, pineapple, apple, brandy	$1.35\times10^{2}\pm9.94$	$3.56\times10^{2}\pm7.07$	↗
Ethyl octanoate-M	ES21	106-32-1	fruity, pineapple, apple, brandy	$3.45\times10^{2}\pm1.45\times10$	$3.73\times10^{2}\pm1.19\times10$	ns

Ethyl pentanoate	ES22	539-82-2	apple, pineapple, green	$5.21\times10\pm5.32$	$4.09\times10^{2}\pm4.39\times10$	↗
Ethyl propanoate	ES23	105-37-3	grape, pineapple, fruity, rum	$1.47\times10^{3}\pm2.56\times10$	$1.71\times10^{3}\pm4.65\times10$	↗
gamma-Butyrolactone	ES24	96-48-0	cream, fat, caramel	$7.53\times10^{2}\pm2.35\times10$	$6.86\times10^{2}\pm3.10\times10$	ns
Hexyl propanoate	ES25	2445-76-3	sweet fruity, earthy, pungent aroma	$1.04\times10^{2}\pm5.74$	$1.61\times10^{2}\pm1.29\times10$	↗
Isoamyl acetate	ES26	123-92-2	sweet, banana, fruity	$7.75\times10^{2}\pm4.40\times10$	$2.21\times10^{3}\pm2.44\times10^{2}$	↗
Methyl 2-methylbutanoate	ES27	868-57-5	apple	$2.23\times10^{2}\pm9.48\times10^{-1}$	$3.01\times10^{2}\pm2.42\times10$	↗
Methyl 3-methylbutanoate	ES28	556-24-1	strong apple, pineapple	$2.63\times10^{2}\pm1.70\times10$	$6.04\times10^{2}\pm9.54\times10$	↗
Methyl acetate	ES29	79-20-9	ethereal	$2.01\times10^{3}\pm1.16\times10^{2}$	$1.91\times10^{3}\pm9.09\times10$	ns
Propyl acetate	ES30	109-60-4	fruity, pear	$2.21\times10^{2}\pm2.97$	$2.84\times10^{2}\pm4.23\times10$	ns
Alcohols						
(Z)-3-Hexenol	AL1	928-96-1	green, herb	$1.14\times10^{2}\pm4.49$	$1.33\times10^{2}\pm3.12$	↗
1-Butanol	AL2	71-36-3	wine	$2.06\times10^{3}\pm2.68\times10$	$4.28\times10^{3}\pm3.69\times10$	↗
1-Hexanol-D	AL3	111-27-3	fresh, fruity, wine, sweet, green	$2.81\times10^{2}\pm2.76$	$4.31\times10^{2}\pm5.13\times10$	↗
1-Hexanol-M	AL4	111-27-3	fresh, fruity, wine, sweet, green	$2.72\times10^{2}\pm4.44$	$1.61\times10^{2}\pm9.90$	↘
1-Pentanol-D	AL5	71-41-0	balsamic	$6.47\times10\pm3.71$	$5.74\times10\pm4.36$	ns
1-Pentanol-M	AL6	71-41-0	balsamic	$2.01\times10^{2}\pm1.39\times10$	$1.42\times10^{2}\pm1.20\times10$	↘
1-Penten-3-ol	AL7	616-25-1	ethereal, green, tropical fruity	$5.25\times10^{2}\pm7.09$	$1.82\times10^{2}\pm7.05$	↘
1-Propanol	AL8	71-23-8	alcohol, pungent	$1.32\times10^{3}\pm3.47\times10$	$1.05\times10^{3}\pm4.61\times10$	↘
2-Butanol	AL9	78-92-2	fruity	$1.66\times10^{3}\pm5.41\times10$	$1.13\times10^{3}\pm4.93\times10$	↘
2-Methyl-1-propanol	AL10	78-83-1	fresh, alcoholic, leather	$6.12\times10^{3}\pm1.09\times10^{2}$	$6.31\times10^{3}\pm9.77\times10$	ns
3-Methyl-1-butanol-D	AL11	123-51-3	whiskey, banana, fruity	$6.23\times10^{3}\pm2.29\times10$	$7.73\times10^{3}\pm4.95\times10$	↗
3-Methyl-1-butanol-M	AL12	123-51-3	whiskey, banana, fruity	$7.05\times10^{2}\pm2.29\times10$	$3.14\times10^{2}\pm8.11\times10^{-1}$	↘
3-Pentanol	AL13	584-02-1		$6.50\times10^{2}\pm2.75\times10$	$6.31\times10^{2}\pm6.32\times10$	ns
cis-2-Penten-1-ol	AL14	1576-95-0	green, plastic, rubber	$2.50\times10^{2}\pm1.07\times10$	$2.02\times10^{2}\pm1.15\times10$	↘
Ethanol	AL15	64-17-5	aromaticity	$8.21\times10^{4}\pm5.95\times10^{2}$	$9.81\times10^{4}\pm2.52\times10^{3}$	↗
Furfuryl alcohol	AL16	98-00-0	bready	$1.67\times10^{3}\pm1.17\times10^{2}$	$3.06\times10^{3}\pm1.74\times10^{2}$	↗

Aldehydes						
(E)-2-Heptenal	AD1	18829-55-5	spicy, green vegetables, fresh, fatty	$5.22\times10\pm1.08\times10$	$2.66\times10\pm1.86$	↘
(E)-2-Octenal	AD2	2548-87-0	fresh cucumber, fatty, green herbal, banana	$5.80\times10\pm9.10$	$5.45\times10\pm2.53$	ns
(E)-2-Pentenal	AD3	1576-87-0	potato, peas	$4.39\times10^{2}\pm2.30\times10$	$1.42\times10^{2}\pm6.75$	↘
2-Methylbutanal	AD4	96-17-3	almond, cocoa, malt	$3.98\times10^{2}\pm1.90\times10$	$2.23\times10^{2}\pm2.96\times10$	↘
2-Methylpropanal	AD5	78-84-2	banana, melon, slightly nutty	$3.10\times10^{2}\pm2.55\times10$	$2.03\times10^{2}\pm5.97$	↘
2-Propenal	AD6	107-02-8	strong pungent	$2.02\times10^{2}\pm2.15\times10$	$1.83\times10^{2}\pm5.69$	ns
3-Methyl-2-butenal-D	AD7	107-86-8	fruity	$6.18\times10^{2}\pm2.32\times10$	$4.77\times10^{2}\pm1.41\times10$	↘
3-Methyl-2-butenal-M	AD8	107-86-8	fruity	$4.68\times10^{2}\pm7.19$	$4.02\times10^{2}\pm1.03\times10$	↘
3-Methylbutanal	AD9	590-86-3	chocolate, fat	$4.10\times10^{2}\pm3.76\times10$	$3.37\times10^{2}\pm2.23\times10$	ns
Acetaldehyde	AD10	75-07-0	green, slight fruity	$1.72\times10^{3}\pm6.17\times10$	$2.23\times10^{3}\pm1.80\times10^{2}$	↗
Furfural-D	AD11	1998-01-1	sweet, woody, almond, bready	$4.86\times10^{2}\pm1.84\times10$	$2.48\times10^{2}\pm8.16$	↘
Furfural-M	AD12	1998-01-1	sweet, woody, almond, bready	$8.93\times10^{2}\pm2.67\times10$	$5.41\times10^{2}\pm3.06\times10$	↘
Hexanal	AD13	66-25-1	fresh, green, fat, fruity	$1.50\times10^{2}\pm8.43\times10^{-1}$	$9.37\times10\pm3.61$	↘
Nonanal	AD14	124-19-6	rose, citrus, strong oily	$1.19\times10^{2}\pm6.65$	$7.33\times10\pm4.78$	↘
Octanal	AD15	124-13-0	aldehyde, waxy, citrus, orange, fruity, fatty	$3.22\times10\pm1.16$	$4.81\times10\pm2.32$	↗
Pentanal	AD16	110-62-3	green grassy, faint banana, pungent	$3.07\times10^{2}\pm5.99$	$1.44\times10^{2}\pm1.50\times10$	↘
Phenylacetaldehyde-D	AD17	122-78-1	hyacinth, sweet fruity, almond, cherry	$3.62\times10^{2}\pm3.01\times10$	$2.16\times10^{2}\pm4.96$	↘
Phenylacetaldehyde-M	AD18	122-78-1	hyacinth, sweet fruity, almond, cherry	$6.99\times10^{3}\pm2.83\times10$	$4.40\times10^{3}\pm1.47\times10^{2}$	↘
Propanal-D	AD19	123-38-6	pungent, green grassy	$1.80\times10^{3}\pm9.73$	$1.32\times10^{3}\pm5.42\times10$	↘
Propanal-M	AD20	123-38-6	pungent, green grassy	$1.45\times10^{3}\pm2.80\times10$	$1.60\times10^{3}\pm4.27\times10$	↗
Ketones						
1-Hydroxy-2-propanone	KT1	116-09-6	pungent, caramel, fresh	$1.06\times10^{2}\pm3.18$	$2.78\times10\pm3.06$	↘
23-Pentanedione	KT2	600-14-6	sweet, cream, caramel, nuts, cheese	$2.10\times10^{3}\pm9.49$	$1.54\times10^{3}\pm9.94\times10$	↘
2-Butanone	KT3	78-93-3	fruity, camphor	$3.36\times10^{3}\pm3.34\times10$	$2.39\times10^{3}\pm6.08$	↘
2-Heptanone-D	KT4	110-43-0	pear, fruity, slight medicinal fragrance	$5.72\times10^{2}\pm1.66\times10$	$5.87\times10^{2}\pm5.91\times10$	ns

2-Heptanone-M	KT5	110-43-0	pear, fruity, slight medicinal fragrance	$2.59\times10^{2}\pm5.94$	$9.31\times10\pm1.15\times10$	↘
2-Pentanone	KT6	107-87-9	acetone, fresh, sweet fruity, wine	$3.70\times10^{3}\pm1.04\times10^{2}$	$3.29\times10^{3}\pm6.74\times10$	↘
3-Hexanone	KT7	589-38-8	fruity, grape, sweet, rum	$1.73\times10^{3}\pm1.19\times10$	$1.72\times10^{3}\pm5.96\times10$	ns
3-Penten-2-one	KT8	625-33-2	fruity, turns into spicy during storage	$8.97\times10^{2}\pm1.07\times10$	$9.66\times10^{2}\pm3.80\times10$	ns
4-Hexen-3-one	KT9	2497-21-4	pungent, spicy	$3.90\times10\pm3.43$	$9.62\times10\pm1.75$	↗
4-Methyl-2-pentanone	KT10	108-10-1	ketone	$4.06\times10^{2}\pm9.59$	$6.14\times10^{2}\pm6.39\times10$	↗
4-Methyl-3-penten-2-one	KT11	141-79-7	spice, earth, green	$1.36\times10^{2}\pm6.05$	$5.58\times10\pm4.65$	↗
6-Methyl-5-hepten-2-one	KT12	110-93-0	citrus, fruity, mouldy, ketone	$6.31\times10^{2}\pm8.32$	$5.32\times10^{2}\pm8.87$	↘
Acetoin-D	KT13	513-86-0	butter, cream	$1.77\times10^{3}\pm7.05\times10$	$1.74\times10^{3}\pm4.72\times10$	ns
Acetoin-M	KT14	513-86-0	butter, cream	$6.87\times10^{2}\pm2.01\times10$	$3.46\times10^{2}\pm1.21\times10$	↘
Acetone	KT15	67-64-1	fresh, apple, pear	$7.36\times10^{3}\pm1.60\times10$	$7.62\times10^{3}\pm9.07\times10$	↗
Acetophenone	KT16	98-86-2	sweet, spicy, almond	$6.88\times10^{2}\pm2.32\times10$	$4.34\times10^{2}\pm1.29\times10$	↘
alpha-Isophorone	KT17	78-59-1	camphor	$4.42\times10^{2}\pm3.43\times10$	$1.15\times10^{3}\pm3.33\times10$	↗
Cyclopentanone	KT18	120-92-3	pleasant	$1.22\times10^{2}\pm2.56$	$4.19\times10^{2}\pm1.44\times10$	↗
Terpenes						
(Z)-beta-Farnesene	TP1	28973-97-9	green, floral	$1.77\times10^{2}\pm3.49\times10$	$2.86\times10^{2}\pm1.32\times10$	↗
18-Cineole	TP2	470-82-6	camphor, refreshing herbal	$3.77\times10^{2}\pm6.81$	$3.31\times10^{2}\pm1.22\times10$	↘
3-Carene	TP3	13466-78-9	citrus, lemon, woody	$2.39\times10^{2}\pm5.64$	$1.27\times10^{2}\pm6.98$	↘
4-Terpinenol	TP4	562-74-3	pepper, woody, moldy soil	$1.11\times10^{3}\pm6.96\times10$	$2.64\times10^{3}\pm3.44\times10^{2}$	↗
alpha-Terpinene	TP5	99-86-5	woody, lemon, citrus	$1.16\times10^{2}\pm4.55$	$1.75\times10^{2}\pm3.54$	↗
beta-Pinene	TP6	127-91-3	resin, green	$5.15\times10^{2}\pm1.27\times10$	$5.08\times10^{2}\pm1.97\times10$	ns
Carvone	TP7	99-49-0	spearmint	$1.91\times10^{3}\pm1.84\times10^{2}$	$3.50\times10^{3}\pm1.81\times10^{2}$	↗
delta-Cadinene	TP8	483-76-1	thyme, herbal, woody	$5.48\times10^{2}\pm9.14\times10$	$5.43\times10^{2}\pm3.62\times10$	ns
gamma-Terpinene	TP9	99-85-4	oil, wood, terpenes, lemon, lime, herbs	$1.57\times10^{2}\pm1.70$	$4.02\times10^{2}\pm7.49$	↗
Isocineole	TP10	470-67-7	cool, camphor, spice	$2.43\times10\pm3.65$	$7.29\times10\pm7.38$	↗
Limonene	TP11	138-86-3	lemon, sweet, orange, pine oil	$2.06\times10^{2}\pm2.03$	$2.64\times10^{2}\pm2.83$	↗

Linalool	TP12	78-70-6	citrus, rose, woody, blueberry	$1.99\times10^{3}\pm5.34\times10$	$2.25\times10^{3}\pm1.95\times10^{2}$	ns
Linalool oxide-D	TP13	60047-17-8	floral	$4.41\times10^{3}\pm4.85\times10^{2}$	$3.61\times10^{2}\pm1.21\times10$	↘
Linalool oxide-M	TP14	60047-17-8	floral	$1.12\times10^{4}\pm3.74\times10^{2}$	$1.80\times10^{3}\pm6.89\times10$	↘
p-Cymene	TP15	99-87-6	fresh citrus terpene woody spice	$7.35\times10\pm4.84$	$1.41\times10^{2}\pm4.81$	↗
Rose oxide	TP16	16409-43-1	green, rose, floral	$9.30\times10\pm2.49$	$6.18\times10\pm6.83\times10^{-1}$	↘
Sulfur compounds						
Allyl propyl sulfide	SF1	27817-67-0	garlic, onion	$4.33\times10^{2}\pm5.80$	$6.26\times10^{2}\pm5.20$	↗
Diallyl sulfide	SF2	592-88-1	garlic	$3.63\times10^{3}\pm9.69\times10$	$1.71\times10^{3}\pm7.93\times10$	↘
Dimethyl sulfide	SF3	75-18-3	cabbage, sulfur, gasoline	$7.02\times10^{2}\pm1.51\times10$	$4.86\times10^{2}\pm4.61$	↘
Dimethyl trisulfide	SF4	3658-80-8	fresh onion, mint, spicy	$1.55\times10^{2}\pm5.93$	$3.29\times10^{2}\pm2.30\times10$	↗
Dipropyl disulfide	SF5	629-19-6	sulfury, onion	$8.86\times10\pm6.20$	$1.60\times10^{2}\pm1.60\times10$	↗
Dipropyl trisulfide	SF6	6028-61-1	garlic	$3.27\times10^{2}\pm4.94\times10$	$2.33\times10^{2}\pm9.39$	↘
Methional-D	SF7	3268-49-3	onion, meat, fruity	$3.38\times10^{2}\pm2.13\times10$	$1.39\times10^{2}\pm1.26\times10$	↘
Methional-M	SF8	3268-49-3	onion, meat, fruity	$2.32\times10^{3}\pm1.06\times10^{2}$	$1.20\times10^{3}\pm3.76\times10$	↘
Acids						
2-Methylpropanoic acid-D	AC1	79-31-2	yogurt, rancid cream	$2.22\times10^{2}\pm1.96\times10$	$3.26\times10^{2}\pm2.10\times10$	↗
2-Methylpropanoic acid-M	AC2	79-31-2	yogurt, rancid cream	$1.31\times10^{3}\pm3.31\times10$	$1.31\times10^{3}\pm7.53\times10$	ns
Acetic acid	AC3	64-19-7	spicy	$1.54\times10^{4}\pm5.60\times10$	$1.58\times10^{4}\pm3.28\times10^{2}$	ns
Butanoic acid-D	AC4	107-92-6	strong acetic acid, cheese, butter, fruity	$2.43\times10^{2}\pm4.31\times10$	$4.11\times10^{2}\pm3.12\times10$	↗
Butanoic acid-M	AC5	107-92-6	strong acetic acid, cheese, butter, fruity	$2.33\times10^{3}\pm2.26\times10^{2}$	$2.42\times10^{3}\pm1.29\times10^{2}$	ns
Pentanoic acid-D	AC6	109-52-4	fatty, decay, slightly sour, slightly sweet	$6.48\times10^{2}\pm5.67\times10$	$6.99\times10^{2}\pm4.43\times10$	ns
Pentanoic acid-M	AC7	109-52-4	fatty, decay, slightly sour, slightly sweet	$1.06\times10^{4}\pm3.53\times10^{2}$	$9.06\times10^{3}\pm3.67\times10^{2}$	↘
Propanoic acid	AC8	1979-09-4	yogurt, vinegar	$4.56\times10^{2}\pm2.39\times10$	$3.54\times10^{2}\pm2.89$	↘

Others						
1-Methyl-1H-pyrrole	OT1	96-54-8	powerful smoky woody herbal	$5.64\times10^{2}\pm3.08\times10$	$1.71\times10^{2}\pm2.35\times10$	↘
2-Isobutyl-3-methoxypyrazine	OT2	24683-00-9	green pepper	$8.72\times10\pm2.53\times10$	$1.57\times10^{2}\pm3.56$	↗
2-Methylfuran	OT3	534-22-5	chocolate, ether like odor	$2.51\times10^{2}\pm2.64\times10$	$1.77\times10^{2}\pm1.73\times10$	↘
2-Pentylfuran	OT4	3777-69-3	bean, fruity, earthy, green, vegetable	$7.40\times10\pm2.56$	$5.59\times10\pm1.93$	↘
2-Propylpyrazine	OT5	18138-03-9	nut, green	$3.95\times10^{2}\pm3.03\times10$	$1.45\times10^{2}\pm6.94$	↘
4,5-Dimethylthiazole	OT6	3581-91-7	green, nut, roast	$6.01\times10\pm5.85\times10^{-1}$	$5.96\times10\pm3.48$	ns
Acetal	OT7	105-57-7	floral, fruity	$1.17\times10^{3}\pm2.61\times10$	$5.85\times10^{2}\pm3.95\times10$	↘
alpha p-Dimethylstyrene	OT8	1195-32-0	citrus, pine	$1.54\times10^{2}\pm6.86$	$2.92\times10^{2}\pm2.36\times10$	↗
Ethylbenzene	OT9	100-41-4	aromatic odor	$2.12\times10^{2}\pm2.03\times10$	$5.35\times10^{2}\pm1.96\times10$	↗
Methyl chavicol	OT10	140-67-0	sweet, green, herbal, fennel	$4.18\times10^{2}\pm2.67\times10$	$2.98\times10^{2}\pm9.12$	↘

* ↗ and ↘ indicate significantly higher or lower relative contents in ICF compared with TOF (FDR-adjusted $p < 0.05$), respectively

# ns indicates no significant difference.

Table S3. VIP values of OPLS-da based on the GC-IMS data.

Compounds	VIP value	Compounds	VIP value	Compounds	VIP value	Compounds	VIP value
gamma-Terpinene	1.11	1-Hexanol-M	1.11	alpha-Terpinene	1.10	1,8-Cineole	1.08
1-Butanol	1.11	4-Hexen-3-one	1.11	Furfural-M	1.10	Ethyl propanoate	1.07
1-Penten-3-ol	1.11	Acetophenone	1.10	Pentanal	1.10	2,3-Pentanedione	1.07
Linalool oxide-M	1.11	3-Methyl-1-butanol-M	1.10	Ethyl pentanoate	1.10	Dipropyl disulfide	1.06
Cyclopentanone	1.11	Hexanal	1.10	Ethyl lactate-M	1.10	Hexyl propanoate	1.06
2-Butanone	1.11	Ethyl hexanoate-M	1.10	Dimethyl trisulfide	1.10	4-Terpinenol	1.06
1-Hydroxy-2-propanone	1.11	1-Methyl-1H-pyrrole	1.10	Methional-M	1.10	3-Methyl-2-butenal-D	1.06
Ethyl isobutyrate	1.11	3-Methyl-1-butanol-D	1.10	Limonene	1.10	cis-2-Penten-1-ol	1.06
Diallyl sulfide	1.11	Ethyl (E)-2-butenoate	1.10	3-Methylbutyl propanoate	1.09	Ethyl acetate	1.05
Ethyl 3-hydroxybutanoate	1.11	Ethyl hexanoate-D	1.10	Methional-D	1.09	3-Methyl-2-butenal-M	1.05
Rose oxide	1.11	3-Carene	1.10	Furfuryl alcohol	1.09	Ethanol	1.04
Ethyl octanoate-D	1.11	2-Heptanone-M	1.10	Ethyl lactate-D	1.09	1-Propanol	1.04
Acetoin-M	1.11	2-Butanol	1.10	Ethyl butanoate	1.09	2-Methylpropanal	1.04
(E)-2-Pentenal	1.11	2-Methylpropyl acetate	1.10	Isocineole	1.09	Methyl 3-methylbutanoate	1.04
alpha-Isophorone	1.11	Furfural-D	1.10	Carvone	1.08	2-Methylpropanoic acid-D	1.03
Allyl propyl sulfide	1.11	Ethylbenzene	1.10	Isoamyl acetate	1.08	Ethyl benzoate	1.02
Ethyl 3-methylbutanoate-D	1.11	Propanal-D	1.10	alpha,p-Dimethylstyrene	1.08	1-Pentanol-M	1.02
Ethyl 3-methylbutanoate-M	1.11	Acetal	1.10	2-Methylbutanal	1.08	4-Methyl-2-pentanone	1.02
Ethyl 2-methylbutanoate	1.11	2-Propylpyrazine	1.10	Propanoic acid	1.08	Butanoic acid-D	1.02
Phenylacetaldehyde-M	1.11	p-Cymene	1.10	Phenylacetaldehyde-D	1.08	1-Hexanol-D	1.01
4-Methyl-3-penten-2-one	1.11	Linalool oxide-D	1.10	Nonanal	1.08	Pentanoic acid-M	1.01
6-Methyl-5-hepten-2-one	1.11	Dimethyl sulfide	1.10	Methyl chavicol	1.08		

Table S4. Concentrations (mmol/g, mean ± sd) of molecules determined by ^{1}H-NMR in the PXDB with traditional open fermentation (TOF) and industrial close fermentation (ICF).

Compounds	Chemical Shift (ppm)	Multiplicity	J_{H-H}, Hz	TOF	ICF	Significant difference (ICF vs. TOF)
**Amino Acids, Peptides and Analogues**						
4-Aminobutyrate	2.286	$-CH_2$ t	7.36	$4.36\times10^{-3}\pm7.39\times10^{-4}$	$7.26\times10^{-3}\pm2.23\times10^{-3}$	ns[#]
Alanine	1.469	$-CH_3$ d	7.24	$2.08\times10^{-2}\pm6.58\times10^{-4}$	$2.54\times10^{-2}\pm2.21\times10^{-4}$	↗[*]
Asparagine	2.932	$-CH_2$ dd	17.12, 4.24	$2.16\times10^{-2}\pm1.57\times10^{-3}$	$2.03\times10^{-2}\pm3.01\times10^{-3}$	ns
Aspartate	2.809	$-CH_2$ dd	17.22, 3.51	$8.76\times10^{-3}\pm1.09\times10^{-3}$	$1.10\times10^{-2}\pm6.36\times10^{-4}$	⬈
Glutamate	2.102	$-CH_2$ m		$3.15\times10^{-2}\pm1.32\times10^{-3}$	$4.10\times10^{-2}\pm1.46\times10^{-3}$	⬈
Glycine	3.557	$-CH_2$ s		$3.29\times10^{-2}\pm4.36\times10^{-3}$	$3.10\times10^{-2}\pm4.52\times10^{-4}$	ns
Isoleucine	1.001	$-CH_3$ t	7.02	$1.05\times10^{-2}\pm3.90\times10^{-4}$	$1.33\times10^{-2}\pm4.59\times10^{-4}$	⬈
Leucine	0.945	$-CH_3$ d	5.80	$2.19\times10^{-2}\pm9.46\times10^{-4}$	$2.73\times10^{-2}\pm6.78\times10^{-4}$	⬈
Lysine	3.009	$-CH_2$ t	7.55	$1.66\times10^{-2}\pm7.73\times10^{-3}$	$2.23\times10^{-2}\pm1.87\times10^{-3}$	ns
Methionine	2.633	$-CH_2$ t	7.75	$8.68\times10^{-3}\pm1.02\times10^{-3}$	$1.15\times10^{-2}\pm8.53\times10^{-4}$	ns
Phenylalanine	7.419	$2\times -CH$ t	7.50	$2.94\times10^{-4}\pm1.15\times10^{-4}$	$1.82\times10^{-4}\pm8.44\times10^{-6}$	↘
Proline	3.356	$-CH_2$ m		$1.02\times10^{-2}\pm4.96\times10^{-4}$	$1.20\times10^{-2}\pm3.57\times10^{-4}$	ns
Pyroglutamate	2.406	$-CH_2$ m		$4.04\times10^{-3}\pm9.86\times10^{-4}$	$3.09\times10^{-3}\pm2.49\times10^{-4}$	ns
Sarcosine	2.747	$-CH_3$ s		$7.77\times10^{-4}\pm5.06\times10^{-4}$	$5.48\times10^{-4}\pm4.21\times10^{-4}$	ns
Serine	3.972	$-CH_2$ dd	15.99, 3.74	$7.03\times10^{-4}\pm1.13\times10^{-4}$	$7.69\times10^{-4}\pm1.26\times10^{-4}$	ns
Threonine	3.593	$-CH$ d	4.83	$1.53\times10^{-2}\pm5.27\times10^{-3}$	$1.26\times10^{-2}\pm1.54\times10^{-4}$	ns
Tyrosine	6.892	$2\times -CH$ m	8.51	$7.53\times10^{-3}\pm4.03\times10^{-4}$	$7.76\times10^{-3}\pm5.83\times10^{-4}$	ns
Valine	0.974	$-CH_3$ d	7.01	$1.40\times10^{-2}\pm3.41\times10^{-4}$	$1.71\times10^{-2}\pm4.81\times10^{-4}$	⬈

**Alcohols**						
Ethanol	1.183	$-CH_3$ t	7.09	$1.44\times10^{-1}\pm3.82\times10^{-3}$	$1.62\times10^{-1}\pm2.16\times10^{-2}$	ns
Glycerol	3.569	2 × $-CH_2$ dd	11.77, 6.55	$1.27\times10^{-1}\pm6.05\times10^{-3}$	$9.30\times10^{-2}\pm8.70\times10^{-3}$	↘
Mannitol	3.853	$-CH_2$ dd	11.8, 2.78	$2.80\times10^{-4}\pm8.70\times10^{-5}$	$4.74\times10^{-4}\pm2.81\times10^{-4}$	ns
Methanol	3.349	$-CH_3$ s		$2.15\times10^{-2}\pm4.15\times10^{-3}$	$1.94\times10^{-2}\pm1.08\times10^{-2}$	↘
Propylene glycol	1.137	$-CH_3$ d	6.45	$3.95\times10^{-2}\pm1.20\times10^{-2}$	$5.00\times10^{-2}\pm2.83\times10^{-3}$	ns
Carbohydrates						
Arabinose	4.518	$-CH$ d	7.81	$2.38\times10^{-2}\pm3.14\times10^{-3}$	$2.06\times10^{-2}\pm5.34\times10^{-4}$	ns
Fructose	3.563	$-CH_2$ m		$9.54\times10^{-3}\pm5.09\times10^{-3}$	$4.52\times10^{-2}\pm2.51\times10^{-3}$	↗
Fucose	5.201	$-CH$ d	3.93	$3.68\times10^{-2}\pm6.27\times10^{-3}$	$2.28\times10^{-2}\pm1.61\times10^{-2}$	ns
Galactose	5.265	$-CH$ d	3.86	$1.61\times10^{-2}\pm7.65\times10^{-4}$	$1.58\times10^{-2}\pm4.92\times10^{-4}$	ns
Glucose	4.645	$-CH$ d	7.95	$1.11\times10^{-1}\pm2.58\times10^{-2}$	$8.99\times10^{-2}\pm1.42\times10^{-2}$	ns
Ribose	4.932	$-CH$ d	6.47	$1.35\times10^{-2}\pm1.11\times10^{-3}$	$1.50\times10^{-2}\pm1.25\times10^{-3}$	ns
Xylose	4.569	$-CH$ d	7.92	$1.75\times10^{-3}\pm2.31\times10^{-4}$	$8.21\times10^{-4}\pm7.81\times10^{-4}$	ns
**Organic Acids**						
3-Methyl-2-oxovalerate	1.085	$-CH_3$ d	7.00	$1.27\times10^{-4}\pm1.17\times10^{-5}$	$2.29\times10^{-4}\pm4.32\times10^{-5}$	↗
Acetate	1.908	$-CH_3$ s		$3.57\times10^{-2}\pm1.10\times10^{-3}$	$3.41\times10^{-2}\pm3.47\times10^{-3}$	ns
Formate	8.443	$-CH$ s		$2.51\times10^{-3}\pm3.98\times10^{-4}$	$3.29\times10^{-3}\pm1.86\times10^{-4}$	ns
Fumarate	6.51	$-CH$ s		$2.61\times10^{-4}\pm4.76\times10^{-5}$	$2.33\times10^{-4}\pm1.58\times10^{-5}$	ns
Isovalerate	0.902	$-CH_3$ d		$7.88\times10^{-5}\pm2.24\times10^{-5}$	$5.95\times10^{-5}\pm2.04\times10^{-5}$	ns
Lactate	4.091	$-CH$ q	20.79, 6.93	$2.07\times10^{-2}\pm6.26\times10^{-3}$	$2.19\times10^{-2}\pm3.72\times10^{-3}$	ns
Malate	2.669	$-CH_2$ dd	15.48, 3.09	$6.21\times10^{-4}\pm7.68\times10^{-5}$	$8.49\times10^{-4}\pm1.24\times10^{-4}$	ns
Malonate	3.131	$-CH_2$ s		$4.60\times10^{-3}\pm1.00\times10^{-3}$	$3.85\times10^{-3}\pm2.75\times10^{-3}$	ns
trans-Aconitate	6.576	$-CH$ s		$3.61\times10^{-4}\pm1.98\times10^{-5}$	$2.77\times10^{-4}\pm8.34\times10^{-6}$	↘
Succinate	2.39	2 × $-CH_2$ s		$9.15\times10^{-3}\pm1.93\times10^{-4}$	$7.55\times10^{-3}\pm4.89\times10^{-4}$	↘

**Nucleotides and Nucleosides**						
Cytidine	7.82	$-CH$ d	7.56	$4.34\times10^{-4}\pm1.30\times10^{-4}$	$2.91\times10^{-4}\pm6.19\times10^{-5}$	ns
Cytosine	5.972	$-CH$ d	7.15	$4.86\times10^{-4}\pm5.57\times10^{-5}$	$3.09\times10^{-4}\pm7.89\times10^{-5}$	ns
Uracil	7.539	$-CH$ d	7.67	$9.38\times10^{-4}\pm1.00\times10^{-4}$	$5.50\times10^{-4}\pm4.26\times10^{-5}$	↘
Uridine	7.859	$-CH$ d	8.08	$6.36\times10^{-4}\pm3.38\times10^{-5}$	$9.20\times10^{-4}\pm4.55\times10^{-5}$	↗
**Miscellaneous**						
Acetoin	1.376	$-CH_3$ d	7.10	$8.30\times10^{-4}\pm4.49\times10^{-4}$	$2.61\times10^{-4}\pm1.43\times10^{-4}$	ns
Acetone	2.224	$2\times-CH_3$ s		$4.09\times10^{-4}\pm1.11\times10^{-4}$	$4.53\times10^{-4}\pm1.14\times10^{-4}$	ns
Betaine	3.89	$-CH_2$ s		$2.03\times10^{-3}\pm6.03\times10^{-4}$	$1.75\times10^{-3}\pm2.15\times10^{-4}$	ns
Choline	3.192	$3\times-CH_3$ s		$4.61\times10^{-3}\pm3.51\times10^{-4}$	$3.94\times10^{-3}\pm1.58\times10^{-4}$	↘
Histamine	7.919	$-CH$ br s		$7.66\times10^{-4}\pm7.33\times10^{-5}$	$6.10\times10^{-4}\pm1.25\times10^{-4}$	ns
Hypoxanthine	8.194	$-CH$ s		$3.83\times10^{-4}\pm4.50\times10^{-5}$	$3.00\times10^{-4}\pm6.38\times10^{-5}$	ns
O-Phosphocholine	3.202	$3\times-CH_3$ s		$1.06\times10^{-2}\pm4.00\times10^{-3}$	$1.02\times10^{-2}\pm1.55\times10^{-3}$	ns
sn-Glycero-3-phosphocholine	3.221	$3\times-CH_3$ s		$3.26\times10^{-2}\pm1.64\times10^{-2}$	$2.45\times10^{-2}\pm1.19\times10^{-2}$	ns
Trigonelline	4.43	$-CH_3$ s		$8.10\times10^{-4}\pm3.38\times10^{-4}$	$5.21\times10^{-4}\pm2.24\times10^{-4}$	ns
myo-Inositol	3.297	$-CH_2$ t	9.37	$1.69\times10^{-3}\pm4.23\times10^{-4}$	$1.91\times10^{-3}\pm2.98\times10^{-4}$	ns

* ↗ and ↘ indicate significantly higher or lower relative contents in ICF compared with TOF (FDR-adjusted $p < 0.05$), respectively

# ns indicates no significant difference.
